# Spiking Patterns in the Globus Pallidus Highlight Convergent Neural Dynamics across Diverse Genetic Dystonia Syndromes

**DOI:** 10.1002/ana.27185

**Published:** 2025-01-30

**Authors:** Ahmet Kaymak, Fabiana Colucci, Mahboubeh Ahmadipour, Nico Golfrè Andreasi, Sara Rinaldo, Zvi Israel, David Arkadir, Roberta Telese, Vincenzo Levi, Giovanna Zorzi, Jacopo Carpaneto, Miryam Carecchio, Holger Prokisch, Michael Zech, Barbara Garavaglia, Hagai Bergman, Roberto Eleopra, Alberto Mazzoni, Luigi M. Romito

**Affiliations:** ^1^ The Biorobotics Institute Scuola Superiore Sant'Anna Pisa Italy; ^2^ Department of Excellence for Robotics and AI Scuola Superiore Sant'Anna Pisa Italy; ^3^ Movement Disorders Department Fondazione IRCCS Istituto Neurologico Carlo Besta Milan Italy; ^4^ Department of Neuroscience and Rehabilitation University of Ferrara Ferrara Italy; ^5^ Department of Neurosurgery Hadassah Medical Center Jerusalem Israel; ^6^ Faculty of Medicine The Hebrew University Jerusalem Israel; ^7^ Department of Neurology Hadassah Medical Center Jerusalem Israel; ^8^ Neurosurgery Department, Functional Neurosurgery Unit Fondazione IRCCS Istituto Neurologico Carlo Besta Milan Italy; ^9^ Department of Pediatric Neuroscience Fondazione IRCCS Istituto Neurologico Carlo Besta Milan Italy; ^10^ Department of Neuroscience University of Padova Padova Italy; ^11^ Institute of Neurogenomics Helmholtz Zentrum München Munich Germany; ^12^ Institute of Human Genetics, School of Medicine Technical University of Munich Munich Germany; ^13^ Institute for Advanced Study Technical University of Munich Garching Germany; ^14^ Unit of Medical Genetics and Neurogenetics Fondazione IRCCS Istituto Neurologico Carlo Besta Milan Italy; ^15^ Department of Medical Neuroscience Institute of Medical Research Israel‐Canada (IMRIC), The Hebrew University‐Hadassah Medical School Jerusalem Israel; ^16^ The Edmond and Lily Safra Center for Brain Sciences The Hebrew University Jerusalem Israel

## Abstract

**Objective:**

Genetic dystonia is a complex movement disorder with diverse clinical manifestations resulting from pathogenic mutations in associated genes. A recent paradigm shift emphasizes the functional convergence among dystonia genes, hinting at a shared pathomechanism. However, the neural dynamics supporting this convergence remain largely unexplored.

**Methods:**

Herein, we analyzed microelectrode recordings acquired during pallidal deep brain stimulation surgery from 31 dystonia patients with pathogenic mutations in the *AOPEP*, *GNAL*, *KMT2B*, *PANK2*, *PLA2G6*, *SGCE*, *THAP1*, *TOR1A*, and *VPS16* genes. We identified 1,694 single units whose activity was characterized by a broad set of neural features.

**Results:**

*AOPEP*, *PANK2*, and *THAP1* displayed higher firing regularity, whereas *GNAL*, *PLA2G6*, *KMT2B*, and *SGCE* shared a large fraction of bursting neurons (> 26.6%), significantly exceeding the rate in other genes. *TOR1A* and *VPS16* genes constituted an intermediate group, bridging these 2 groups, due to having the highest degree of spiking irregularity. Hierarchical clustering algorithms based on these dynamics confirmed the results obtained with first‐order comparisons.

**Interpretation:**

Despite lacking common molecular pathways, dystonia genes share largely overlapping structures of neural patterns, in particular the degree of pallidal spiking regularity and bursting activity. We propose that the degree of desynchronization facilitated by pallidal neural bursts may explain the variability in deep brain stimulation (DBS) of the globus pallidus internus (GPi) surgery outcomes across genetic dystonia syndromes. Lastly, investigating the effects of genetic mutations on low‐frequency pallidal activity could optimize personalized adaptive DBS treatments in patients with genetic dystonia. ANN NEUROL 2025;97:826–844

Dystonia is a movement disorder marked by persistent or sporadic muscle contractions resulting in abnormal and often repetitive movements, postures, or both.^1^ The inherited forms of dystonia arise from pathological mutations in causative genes with diverse clinical presentations.^1^, ^2^, ^3^ Deep brain stimulation (DBS) of the globus pallidus internus (GPi) is the most effective treatment for medically refractory dystonia.^4^ Intraoperative use of microelectrode recordings (MERs) remains a common clinical procedure for validating the DBS lead location. These recordings are widely utilized for examining the pathophysiology of movement disorders in different brain regions.^5^, ^6^, ^7^, ^8^, ^9^ However, electrophysiological characterization of dystonia genes has been predominantly confined to case studies.^10^, ^11^, ^12^, ^13^

Despite substantial progress made in identifying dystonia‐related genes, understanding the pathogenesis of dystonia remains challenging. Dystonia is hypothesized to be a network disorder involving the cortico‐striate‐thalamus‐cortical network and the cerebellum‐thalamus‐cortical pathway.^14^ The hypothesis proposes that dystonia arises due to aberrant sensorimotor integration within the basal ganglia thalamocortical circuit and is contingent on the specific nuclei, including the globus pallidus.^15^, ^16^ Hence, translating the functional implications of dystonia gene mutations into molecular, cellular, and brain circuitry alterations posits challenges.^17^, ^18^

Genetic dystonia research has explored biochemical mechanisms shared between genes at different scales. For instance, genes linked to DOPA‐responsive dystonia syndromes were proven to be involved in dopamine synthesis and metabolism.^19^ The discovery of various genetic defects converging on abnormal EIF2α pathway activation and disrupted cAMP metabolism in striatal neurons^18^ underscores the relevance of the “convergence” paradigm for dystonia pathogenesis at the molecular level.^17^, ^20^ The same paradigm is further supported by the functional convergence of various dystonia genes into specific neurons at the cellular level.^21^ Similarly, common electrophysiological patterns among dystonia genes are suggested to indicate a shared mechanism at the level of neural circuits.^10^, ^22^ Hence, identifying shared and distinct spiking patterns is crucial for our understanding of genetic dystonia pathophysiology, as these patterns are influenced by molecular changes caused by mutations and they impact the functioning of associated neural circuitries and clinical phenotype.^23^

Herein, we systematically analyzed the pallidal neurons of 31 patients with inherited dystonia (Table 1), encompassing nine dystonia genes (*AOPEP*, *KMT2B*, *GNAL*, *PANK2*, *PLA2G6*, *SGCE*, *THAP1*, *TOR1A*, and *VPS16*),^2^, ^24^, ^25^ from MERs performed during the GPi‐DBS surgery. We aimed to (1) characterize neural activity in the globus pallidus associated with dystonia genes using a broad set of neural features, (2) identify neural dynamics leading to functional convergence/divergence among dystonia genes, and (3) assess the possibility of discriminating dystonia genes via machine learning algorithms. Our findings suggest that dystonia genes may converge at the neural circuitry level through similar pallidal neural signatures, and we propose that examining the impact of genetic mutations on low‐frequency pallidal activity could pave the way for optimized personalized adaptive DBS treatments. We suggest that the degree of desynchronization, driven by varying levels of pallidal neural bursts observed across dystonia genes, may explain the variability in responses to GPi‐DBS treatment among genetic dystonia syndromes.

**Table 1:** Genetic and Clinical Data of the Enrolled Patients

ID	Gene	Variant	Pathogenicity Classification	Phenotype	Main Body Part Affected	Pattern Movements
Pat1	*VPS16*	c.2170_2171delAA (p.Lys724Glufs*44)	Likely pathogenic	M	CR	T
Pat2	*VPS16*	c.2140C>T; p.Gln714*	Likely pathogenic	M	C	P
Pat3	*SGCE*	c.709T>C; p.Arg237*	Pathogenic	M	C	T
Pat4	*VPS16*	c.1939C>T, p.Arg647*	Pathogenic	M	T	T
Pat5	*VPS16*	c.1939C>T, p.Arg647*	Pathogenic	F	CR	T
Pat6	*AOPEP*	c.1909G>T (p.Glu637*)	Likely pathogenic	F	C	T
Pat7	*SGCE*	c.771‐772delAT; p.Cys258*	Pathogenic	M	U	P
Pat8	*THAP1*	c.70_71+8del10 (p.Gly24fs*71 exon 1)	Likely pathogenic	M	C	T
Pat9	*THAP1*	c.70_71+8del10 (p.Gly24fs*71 exon 1)	Likely pathogenic	F	T	T
Pat10	*THAP1*	c.464 A>C (p.Gln155Pro exon 3)	VUS	F	C	T
Pat11	*THAP1*	c.238 A>G (p.Ile80Val exon 2)	Likely pathogenic	F	C	T
Pat12	*PANK2*	c.965A>G (p.Glu322Gly) in exon 2 and c.1561G>A (p.Gly521Arg) in exon 6	Exon.2:likely pathogenic Exon.6: pathogenic	F	CR	T
Pat13	*KMT2B*	c.4561 C>T p.Arg1521Trp	VUS	F	T	T
Pat14	*TOR1A*	c.904906delGAG, p.del302Glu	Pathogenic	F	L	T
Pat15	*TOR1A*	c.904906delGAG, p.del302Glu	Pathogenic	F	L	T
Pat16	*TOR1A*	c.904906delGAG, p.del302Glu	Pathogenic	F	L	T
Pat17	*TOR1A*	c.904906delGAG, p.del302Glu	Pathogenic	F	U	T
Pat18	*TOR1A*	c.904906delGAG, p.del302Glu	Pathogenic	F	C	T
Pat19	*TOR1A*	c.904906delGAG, p.del302Glu	Pathogenic	F	L	T
Pat20	*TOR1A*	c.904906delGAG, p.del302Glu	Pathogenic	F	U	T
Pat21	*TOR1A*	c.904906delGAG, p.del302Glu	Pathogenic	M	U	P
Pat22	*TOR1A*	c.904906delGAG, p.del302Glu	Pathogenic	F	T	T
Pat23	*KMT2B*	c.2240 A>G (p.Gln747Arg)	VUS	F	U	P
Pat24	*SGCE*	c.1037+3delGTGA	Likely Pathogenic	M	U	P
Pat25	*SGCE*	c.233‐I G>T	Likely Pathogenic	M	U	P
Pat26	*GNAL*	c.462_463delGA (p. Lys155Asnfs*9)	Pathogenic	F	C	P
Pat27	*GNAL*	c.628G>A (p.Asp210Asn)	VUS	M	C	P
Pat28	*PANK2*	c.821‐822delCT; p.Leu275Valfs*16 in exon2 and c.1561G>A; p.Gly521Arg in exon 6	Exon2:likely pathogenic Exon 6:pathogenic	F	T	T
Pat29	*PANK2*	c.1213T>G (p.Tyr405Asp) ex.3 and heterozygous deletion of exons 2, 3, 4	Pathogenic	F	T	T
Pat30	*VPS16*	c.290T>A; p.Leu97Gln	VUS	M	U	P
Pat31	*PLA2G6*	p.Ala516Val	Likely pathogenic	F	T	T

The table reports the clinical and genetic data of the enrolled patients. Variant classifications of mutations were made according to Franklin and ACMG criteria (VUS = variant of unknown significance). The table also shows the dystonia phenotype divided into mobile (M) or fix (F), the main body part affected by dystonia (CR = cranial; C = cervical; T = trunk; U = upper limbs; and L = lower limb) and the pattern of dystonic movements (T = tonic and P = phasic) presented by patients.

## Methods

### Study Design and Patient Recruitment

We conducted a retrospective cohort study with patients with genetic dystonia who underwent bilateral GPi‐DBS surgery at the Fondazione IRCCS Istituto Neurologico Carlo Besta (INCB) between January 1999 and December 2021. Patients were considered for DBS surgery based on the INCB criteria: exhibiting generalized or focal dystonia as the primary motor symptom causing severe disability and showing either an unsatisfactory response or intolerance to medical treatment. Those with medical contraindications to surgery, severe psychiatric issues, or an inability to adhere to the surgical care program were deemed ineligible for the surgery. We considered patients with a minimum follow‐up period of 1 year and the availability of high‐quality intraoperative neuronal recordings for further analyses. Patients with acquired or idiopathic dystonia and those with poor‐quality intraoperative recordings, preventing any data extrapolation, were excluded from the study. The demographic and clinical profiles of the patients are reported in Tables 1 and 2.

**Table 2:** Demographic and Surgical Data of the Enrolled Patients

ID	Gene	Sex	Onset Age	Surgery Age	BFMDRS‐M Pre‐DBS*/128	BFMDRS‐M 1 Y‐FU*/128	BFMDRS‐M 3 Y‐FU*/128	BFMDRS‐M 5 Y‐FU*/128	Anesthetics (remifentanil/propofol)
Pat1	*VPS16*	F	12	23	23.0	13.5	19.5	18.5	0.8 / 1.5
Pat2	*VPS16*	M	15	43	32.0	15.5	18.5	17.5	0.5 / 2.0
Pat3	*SGCE*	F	2	33	54.5	19.0	9.0	15.5	0.5 / 1.5
Pat4	*VPS16*	M	14	49	52.5	22.5	9.0	11.0	0.8 / 1.8
Pat5	*VPS16*	F	13	41	88.0	28.0	20.0	23.0	0.6 / 1.5
Pat6	*AOPEP*	M	12	20	63.0	65.0	38.5	40.0	0.5 / 1.7
Pat7	*SGCE*	F	10	20	12.0	8.0	9.0	8.0	0.6 / 1.8
Pat8	*THAP1*	F	40	48	17.0	20.0	16.0	18.0	0.4 / 1.5
Pat9	*THAP1*	F	7	17	32.5	5.0	5.5	7.0	0.5 / 1.2
Pat10	*THAP1*	M	6	14	45.0	20.0	23.0	23.0	0.4 / 1.6
Pat11	*THAP1*	M	14	32	60.5	31.0	26.5	22.0	0.6 / 1.8
Pat12	*PANK2*	F	16	25	89.5	80.0	76.0	76.0	0.5 / 2.0
Pat13	*KMT2B*	M	28	45	27.0	10.0	13.5	8.5	0.3 / 2.0
Pat14	*TOR1A*	M	12	45	39.0	5.0	8.5	8.0	0.0 / 0.0*
Pat15	*TOR1A*	M	13	43	29.5	4.0	14.0	16.0	0.5 / 2.0
Pat16	*TOR1A*	F	7	9	43.0	4.0	4.0	4.0	0.5 / 1.7
Pat17	*TOR1A*	M	10	49	25.0	18.0	na	na	0.5 / 1.9
Pat18	*TOR1A*	M	12	33	36.0	19.0	9.0	8.0	0.0 / 1.5
Pat19	*TOR1A*	M	10	47	23.5	10.0	19.0	9.0	0.4 / 1.3
Pat20	*TOR1A*	F	11	44	59.0	17.5	19.5	11.0	0.0 / 0.0*
Pat21	*TOR1A*	M	14	46	30.5	24.5	9.0	NA	0.7 / 1.0
Pat22	*TOR1A*	M	8	9	34.0	3.0	6.0	NA	0.7 / 1.1
Pat23	*KMT2B*	M	8	16	28.0	14.0	17.0	15.0	0.6 / 0.5
Pat24	*SGCE*	M	2	29	11.5	10.5	NA	NA	0.4 / 1.5
Pat25	*SGCE*	M	3	14	16.0	2.5	NA	NA	0.5 / 0.8
Pat26	*GNAL*	F	37	42	26.0	22.0	20.0	13.0	0.3 / 1.6
Pat27	*GNAL*	F	42	63	24.0	14.0	16.0	13.0	0.6 / 2.0
Pat28	*PANK2*	F	2	10	78.5	74.5	78.5	70.5	0.1 / 2.0
Pat29	*PANK2*	M	2	6	96.0	80.0	77.0	NA	0.7 / 1.3
Pat30	*VPS16*	M	11	19	25.0	19.0	19.0	34.0	0.8 / 1.2
Pat31	*PLA2G6*	F	28	34	66.5	60.5	58.5	NA	0.4 / 2.0

The table reports the demographic and genetic data of the enrolled patients. In addition, data on the motor component of the Burke‐Fahn‐Marsden Dystonia Rating Scale (BFMDRS) preimplant and 1, 3, and 5 years following deep brain stimulation (DBS) surgery are reported. The following data on the medications used during anesthesia were reported: most patients received general anesthesia comprising propofol (mg/kg/h) and/or remifentanil (μg/kg/min).

FU = follow‐up; NA = not applicable.

* Only 2 rounds of general anesthesia were achieved with 1 μg/kg/h of dexmedetomidine.

The study was approved by the Ethical Committee of INCB. Informed consent was obtained from patients and their legal representatives.

### Genetic Screening and Variant Confirmation

Different genetic tests were performed on all patients at different time points. Before 2000, only *TOR1A* was detected in patients. Between 2000 and 2015, individuals who initially tested negative for the *TOR1A* pathogenic variant were retested using Sanger sequencing, as new genes were identified. From 2015 onward, an next‐generation sequencing (NGS)‐customized gene panel for dystonia was applied.^26^ In patients whose rescreening was negative and the clinical phenotype indicated inherited dystonia, whole‐exome sequencing was conducted. Among the enrolled patients, 5 were identified with “variants of unknown significance” (VUS). The causative genes of the patients along with the variant type and associated pathogenicity scores are presented in Table 1.

### Surgical Procedure

Surgeries were performed bilaterally and under stereotactic conditions with the Leksell (Elekta) or Maranello (Eidos22) frame. Our routine surgical procedure has been extensively discussed elsewhere.^27^

The reconstruction of GPi, globus pallidus externa (GPe), and recording depths are defined using the Distal Atlas^28^ in Montreal Neurological Institute (MNI) space (*p* > 0.5 thresholds for definitions of nuclei borders) by Lead DBS^29^ version 2.5.2 suite and using postoperative brain magnetic resonance imaging (MRI) or computed tomography (CT) images (Supplementary Fig S1). Recordings are visually controlled by an expert electrophysiologist.

### Microelectrode Recording Processing

After performing the MER, we used a semi‐parametric offline spike sorting algorithm^30^ to isolate single‐unit activities (SUAs; Supplementary Section 1). We analyzed the SUAs in the temporal and frequency domains and extracted a wide range of features, including spiking patterns, firing rate, regularity, burstiness, oscillations, and neural pauses (Supplementary Section 2, Supplementary Figs S2 and S3). This feature extraction schema has been previously used in the study of neural dynamics within various stimulation targets for other movement disorders.^7^, ^8^, ^31^

The MATLAB source code for our neural feature extraction library is available at github.com/ahmetofficial/Spike-Feature-Generator/.

### Statistical Analyses

Multiple statistical tests were used to compare neural features among genes. We utilized the Kruskal–Wallis test with the post hoc Mann–Whitney *U* test for continuous features. We performed the chi‐square (*χ* ^2^) test and Fisher's Exact test on binary features. For all the statistical analyses, *p* values were corrected either with the Holm–Bonferroni or Benjamini–Hochberg (false discovery rate [FDR]) correction methods, and *p* ≤ 0.05 was considered to indicate statistical significance.

To capture meaningful variations between our neuron populations, we computed effect sizes only when statistical significance was observed.^32^ For Kruskal–Wallis and Mann–Whitney *U* tests, we measured Cohen's d^33^ effect size. We utilized Cramer's v effect size for the chi‐square test and the odds ratio for the Fisher Exact test^33^ (Supplementary Section 3).

### Principal Component Analysis

We performed principal component analysis (PCA) to assess potential separation among our neuron populations based on variance structure. To measure the degree of separability between our neuron populations, we used the Calinski–Harabasz score, which evaluates cluster separation quality by comparing intercluster variance to intracluster variance.^34^

### Gene Stratification by Thresholding

We created a framework to stratify the pallidal neural activities of dystonia genes by assessing the fraction of neurons displaying a particular neural dynamic (neural bursts, oscillations, etc.). To assess whether the observed rate of dynamic expression was lower for one gene than for another, we conducted one‐sided Fisher Exact tests. The test results revealed overlaps and differences among genes, which led to the formation of distinct groups. Subsequently, we concentrated on adjacent group pairs to establish statistically defined thresholds. For each pair, we selected two genes: the gene with the lowest fraction in the upper group (high boundary gene) and the gene with the highest fraction in the lower group (low boundary gene). To estimate the threshold, we incrementally increased the fraction of the low boundary gene, conducting one‐sided Fisher Exact tests at each step until statistical significance was no longer observed between boundary genes (Supplementary Figs S4A, S4B).

### Gene Stratification by Distance

We additionally stratified dystonia genes using the Jensen–Shannon distance^35^ of features representing discharge patterns, firing regularity, neural bursts, neural oscillations, and neural pauses (Supplementary Table S7). This process resulted in a distance matrix among genes for each feature. We aggregated these distance matrices to create a cumulative distance matrix for the selected phenomena. We then performed agglomerative clustering with the Unweighted Pair Group Method with the Arithmetic Mean^36^ (UPGMA) algorithm, a common technique used for distance matrices, on cumulative distance matrices to mathematically cluster these genes. We presented the results of this hierarchical clustering method with dendrograms (Supplementary Fig 4C).

### Neural Decoding for Genetic Dystonia Syndromes

We translated neurophysiological characterizations of dystonia genes into practical use by developing two machine learning pipelines, one‐versus‐one (OVO) and one‐versus‐rest (OVR), for decoding pallidal neurons (Supplementary Section 5).

## Results

### Pallidal Discharge Patterns Differ Significantly Across Dystonia Genes

We initiated the characterization of the neural activity in the globus pallidus across different dystonia genes by measuring and comparing the fractions of neurons exhibiting tonic, irregular, and bursting dynamics (see the Methods section).

**FIGURE 1:**
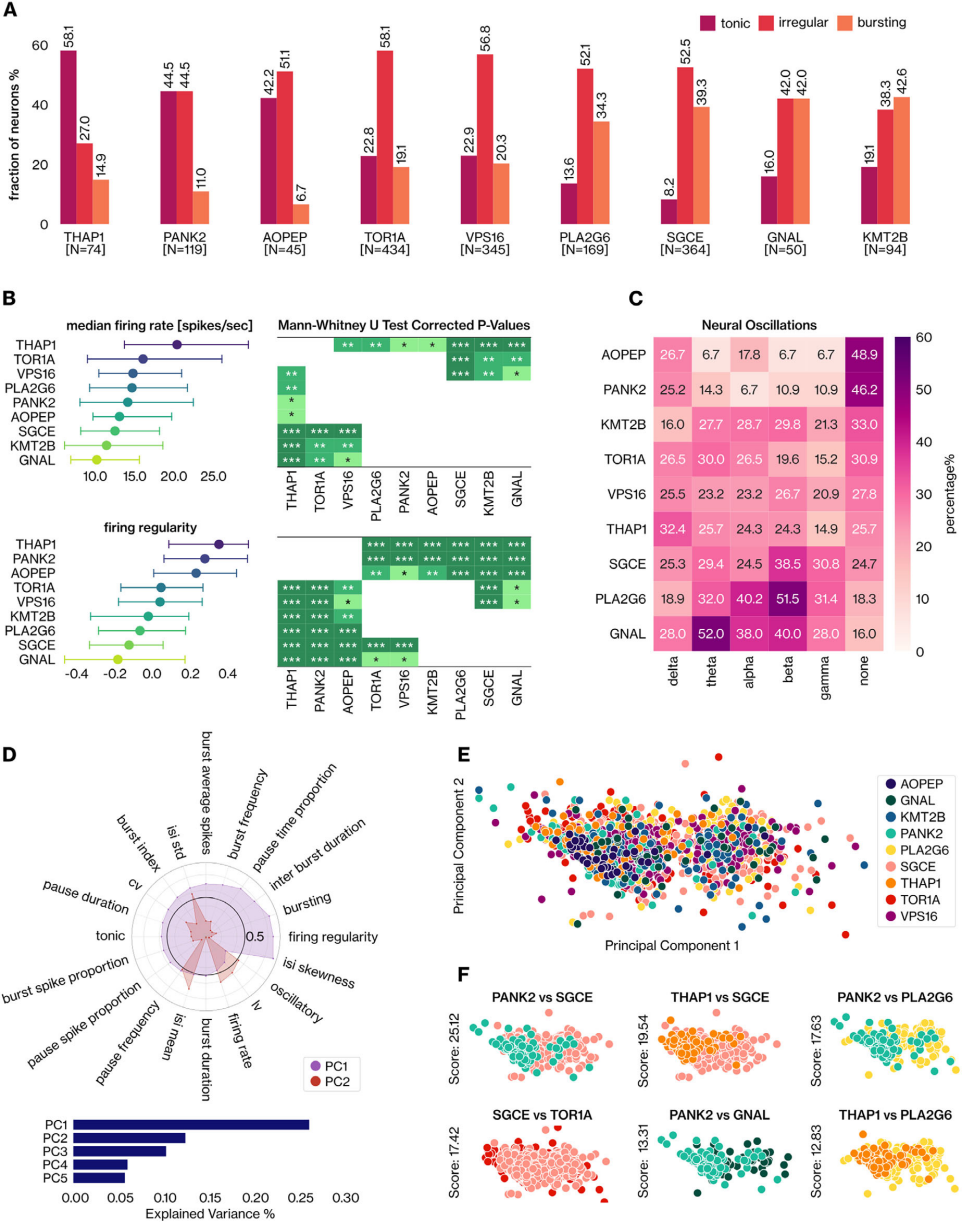
Dystonia genes show diverse neural activity and variance structure within the globus pallidus. (A) The fraction of tonic, irregular, and bursting neurons for dystonia genes, along with the corresponding number of neurons, is presented. (B) The median values and interquartile ranges for firing rate (*top*, *left*) and firing regularity (*bottom*, *left*) are shown. The nonparametric comparison of these features was performed with the Mann–Whitney *U* test with Holm–Bonferroni correction (*p* < 0.05). (C) The fraction of neurons of dystonia genes exhibiting significant neural oscillations in the delta [1–4 Hz], theta [4–8 Hz], alpha [8–12 Hz], beta [12–30 Hz], and gamma [30–100 Hz] bands. (D) Neural features having a minimum loading of 0.5 (in absolute values) on either the first or second principal component (PC) (*top*) and the capacity of the first five principal components to explain total variance in neural activity (*bottom*). (E) The projection of the pallidal activity from the high‐dimensional feature space onto a two‐dimensional PC space. (F) The distance between the variance structures of the gene pairs in the first two PCs is reported with Calinski–Harabasz (CH) scores. [Color figure can be viewed at www.annalsofneurology.org]

The analysis revealed three macro groups among dystonia genes. A large proportion of *THAP1*, *PANK2*, and *AOPEP* neurons showed tonic firing. Neurons displaying irregular spiking behavior comprised ≥ 50% for *AOPEP*, *TOR1A*, *VPS16*, *SGCE*, and *PLA2G6*. Finally, *GNAL*, *SGCE*, *PLA2G6*, and *KMT2B* genes were distinguished by a fraction of neurons exhibiting bursting activity exceeding 30% (Fig 1A). We then compared the neural activity across dystonia genes, considering a broad spectrum of features (Table 3). The median firing rate varied within a narrow range where *GNAL*, *KMT2B*, and *SGCE* neurons displayed significantly reduced firing activity compared with *THAP1*, *TOR1A*, and *VPS16* neurons (*p* ≤ 0.05, Mann–Whitney *U* test with Holm‐Bonferroni correction; Fig 1B, top). Firing regularity varied significantly (Fig 1B, bottom left) across genes, and we identified two distinct groups *THAP1*, *PANK2*, and *AOPEP* and *PLA2G6*, *SGCE*, and *GNAL* that significantly differed (*p* ≤ 0.05; Fig 1B, bottom). Similar results were achieved considering other regularity metrics, such as the coefficient of variation (CV) and asymmetry index. We also analyzed the properties of neural bursts. Interestingly, *PANK2* and *AOPEP* displayed low participation to burst (see Fig 1A), however, they presented the longest burst duration (0.29 seconds), and the most intense bursts (mean burst frequency of 208.1 ± 103.2 hertz [Hz]), respectively. *GNAL*, *PLA2G6*, and *SGCE* neurons are predominantly silent, when observed, with pauses accounting for more than half of their activity (pause time proportion ≥ 50%). The majority of *GNAL* (84%) and *PLA2G6* (81.7%) neurons presented significant oscillations in at least one of the five physiological frequency bands. In contrast, *AOPEP* and *PANK2* neurons displayed weaker oscillatory tendencies, with approximately half of them showing oscillations, mainly in the delta band (Fig 1C).

**Table 3:** Summary of Globus Pallidus Neural Patterns Observed for Dystonia‐Causing Genes

Neural Feature	*AOPEP*	*GNAL*	*KMT2B*	*PANK2*	*PLA2G6*	*SGCE*	*THAP1*	*TOR1A*	*VPS16*
Firing rate (spikes/sec)	15.35 ± 6.9	13.41 ± 9.73	16.71 ± 21.76	20.3 ± 26.36	16.73 ± 10.59	14.95 ± 11.96	23.96 ± 17.74	20.21 ± 15.46	17.15 ± 10.99
Firing regularity	0.23 ± 0.32	−0.13 ± 0.46	−0.02 ± 0.43	0.32 ± 0.49	−0.02 ± 0.49	−0.14 ± 0.36	0.33 ± 0.4	0.06 ± 0.4	0.06 ± 0.47
Coefficient of variation	1.14 ± 0.34	1.82 ± 0.8	1.6 ± 1.1	1.18 ± 0.64	1.59 ± 0.77	1.77 ± 1.07	1.45 ± 1.12	1.49 ± 0.85	1.51 ± 0.98
Local variation	0.72 ± 0.15	0.79 ± 0.24	0.81 ± 0.25	0.69 ± 0.23	0.75 ± 0.22	0.82 ± 0.23	0.62 ± 0.16	0.78 ± 0.22	0.77 ± 0.19
ISI mean (ms)	78.08 ± 31.98	116.25 ± 77.52	117.18 ± 91.53	91.23 ± 68.5	89.38 ± 66.0	96.52 ± 58.25	62.93 ± 44.84	82.49 ± 64.41	78.93 ± 46.01
ISI std (ms)	73.12 ± 37.73	134.28 ± 107.47	128.45 ± 111.73	84.74 ± 72.9	95.99 ± 82.11	108.96 ± 74.33	56.4 ± 46.15	87.91 ± 85.91	83.2 ± 63.71
ISI skewness	1.8 ± 0.3	2.19 ± 0.49	2.07 ± 0.44	1.75 ± 0.38	2.07 ± 0.42	2.18 ± 0.39	1.73 ± 0.35	1.98 ± 0.38	1.99 ± 0.43
ISI corr. coefficient	0.13 ± 0.14	0.02 ± 0.1	0.04 ± 0.15	0.12 ± 0.2	0.04 ± 0.12	0.05 ± 0.11	0.12 ± 0.17	0.05 ± 0.12	0.06 ± 0.13
Asymmetry index	0.36 ± 0.19	0.17 ± 0.2	0.15 ± 0.16	0.33 ± 0.32	0.21 ± 0.21	0.17 ± 0.21	0.34 ± 0.26	0.22 ± 0.27	0.23 ± 0.28
Burst index	20.63 ± 15.63	31.6 ± 26.26	24.96 ± 26.92	25.09 ± 34.62	18.29 ± 25.71	24.05 ± 25.68	8.34 ± 5.93	28.82 ± 28.41	22.42 ± 19.42
Burst duration (sec)	0.11 ± 0.1	0.2 ± 0.18	0.17 ± 0.25	0.29 ± 0.29	0.12 ± 0.12	0.13 ± 0.18	0.17 ± 0.24	0.21 ± 0.35	0.13 ± 0.17
Burst frequency (Hz)	208.1 ± 103.23	116.8 ± 60.91	130.3 ± 83.52	100.28 ± 88.02	141.29 ± 71.41	142.58 ± 76.78	144.22 ± 95.62	137.48 ± 80.32	151.7 ± 80.46
Interburst duration (sec)	3.01 ± 0.49	3.89 ± 2.07	4.58 ± 3.61	3.11 ± 1.4	3.05 ± 1.62	3.52 ± 1.84	2.14 ± 0.73	3.28 ± 1.67	3.12 ± 1.7
Burst count	1.33 ± 0.58	4.48 ± 4.55	3.85 ± 3.79	3.0 ± 1.78	5.28 ± 4.52	4.55 ± 3.89	4.18 ± 2.09	4.0 ± 6.98	3.19 ± 2.49
Burst average spike count	12.17 ± 10.75	10.28 ± 5.61	10.13 ± 13.89	11.08 ± 10.17	9.23 ± 6.6	9.09 ± 7.45	10.33 ± 4.48	11.08 ± 7.73	9.71 ± 7.29
Burst spike proportion (%)	5.41 ± 4.35	3.55 ± 3.69	3.0 ± 3.72	5.81 ± 6.37	3.25 ± 3.38	2.57 ± 2.8	2.94 ± 1.78	3.73 ± 3.4	4.03 ± 4.09
Pause index	0.95 ± 0.59	2.04 ± 6.08	1.39 ± 1.73	1.38 ± 1.5	1.58 ± 5.03	1.18 ± 3.65	1.03 ± 2.21	0.99 ± 1.94	1.03 ± 2.8
Pause ratio	0.14 ± 0.11	0.5 ± 1.5	0.37 ± 0.73	0.28 ± 0.45	0.36 ± 1.26	0.27 ± 0.91	0.17 ± 0.4	0.19 ± 0.3	0.19 ± 0.61
Pause duration (sec)	0.52 ± 0.43	1.05 ± 0.99	0.81 ± 0.96	0.55 ± 0.68	0.67 ± 1.04	0.75 ± 0.82	0.45 ± 0.38	0.62 ± 0.75	0.65 ± 0.98
Pause frequency (Hz)	3.42 ± 1.77	2.41 ± 1.74	3.4 ± 4.43	3.87 ± 3.3	3.22 ± 2.18	2.87 ± 2.23	4.9 ± 3.92	4.22 ± 3.42	3.61 ± 2.39
Pause count	5.02 ± 2.94	7.92 ± 6.29	12.9 ± 15.55	5.6 ± 5.63	10.88 ± 8.76	9.82 ± 7.13	5.51 ± 3.84	8.39 ± 6.85	7.37 ± 4.62
Pause spike proportion %	7.29 ± 2.79	9.48 ± 4.29	9.2 ± 3.89	6.8 ± 3.34	9.5 ± 3.77	10.03 ± 4.2	6.02 ± 3.29	8.19 ± 3.42	8.62 ± 4.03
Pause time proportion %	35.28 ± 15.78	55.27 ± 23.15	47.5 ± 22.61	32.64 ± 18.63	52.39 ± 24.8	52.63 ± 23.3	31.49 ± 20.6	41.62 ± 20.5	43.79 ± 22.75
Delta band frequency (Hz)	0.92 ± 0.0	1.25 ± 0.71	1.01 ± 0.11	0.93 ± 0.05	1.04 ± 0.28	1.04 ± 0.32	0.92 ± 0.0	1.0 ± 0.35	0.96 ± 0.25
Theta band frequency (Hz)	3.66 ± 0.0	4.87 ± 1.44	4.21 ± 1.02	3.83 ± 0.55	4.46 ± 1.16	4.42 ± 1.19	3.8 ± 0.51	4.12 ± 0.9	4.19 ± 0.95
Alpha band frequency (Hz)	8.79 ± 1.66	8.33 ± 1.1	8.76 ± 1.34	8.51 ± 1.61	9.11 ± 1.38	8.86 ± 1.26	8.63 ± 1.52	8.66 ± 1.33	8.67 ± 1.33
Beta band frequency (Hz)	14.65 ± 5.07	16.48 ± 4.48	16.74 ± 5.56	20.4 ± 5.02	17.51 ± 4.82	18.82 ± 5.02	15.14 ± 3.79	17.23 ± 5.84	17.8 ± 5.37
Gamma band frequency (Hz)	52.25 ± 10.99	41.12 ± 14.3	41.46 ± 17.0	46.09 ± 17.64	36.48 ± 7.04	41.73 ± 15.38	34.76 ± 6.81	41.46 ± 15.89	39.73 ± 13.71

The values are indicated as the mean ± standard deviation.

Statistics on neural bursts and oscillation‐related features pertain exclusively to bursting and oscillatory neurons, respectively.

Overall, these results indicate that firing patterns, more than firing intensity, display reliable differences between dystonia genes in the globus pallidus. Firing activity of dystonia genes inside the globus pallidus can be clustered into three main groups: (1) genes associated with high regularity, weak bursts, and oscillations (eg, *AOPEP*); (2) genes associated with low regularity, strong bursts, and oscillations (eg, *PLA2G6*); and (3) intermediate genes (eg, *VPS16*).

We further discriminated dystonia genes regarding their variance structure by PCA (see the Methods section). The first and second principal components (PCs) accounted for 26.21% and 12.43% of the total variance, respectively (Fig 1D, bottom). We projected the pallidal activity of dystonia genes from a high‐dimensional feature space onto a two‐dimensional PC space to represent neurons under maximum variance conditions (Fig 1E). We measured the Calinski–Harabasz (CH) scores to quantify the distances between the neuron populations. *PANK2* and *SGCE* neurons presented the highest level of separability, followed by the *THAP1*‐*SGCE* and *PANK2*‐*PLA2G6* pairs (Fig 1F, top row). In contrast, the variance structures of *GNAL*‐*KMT2B* and *TOR1A*‐*VPS16* were mostly similar. The variance analysis of neural features affirmed the distinctions of *AOPEP*, *PANK2*, and *THAP1* genes from the others (see Fig 1F).

### Neural Bursts and Spiking Regularity Support Functional Convergence of Dystonia Genes in the Globus Pallidus

**FIGURE 2:**
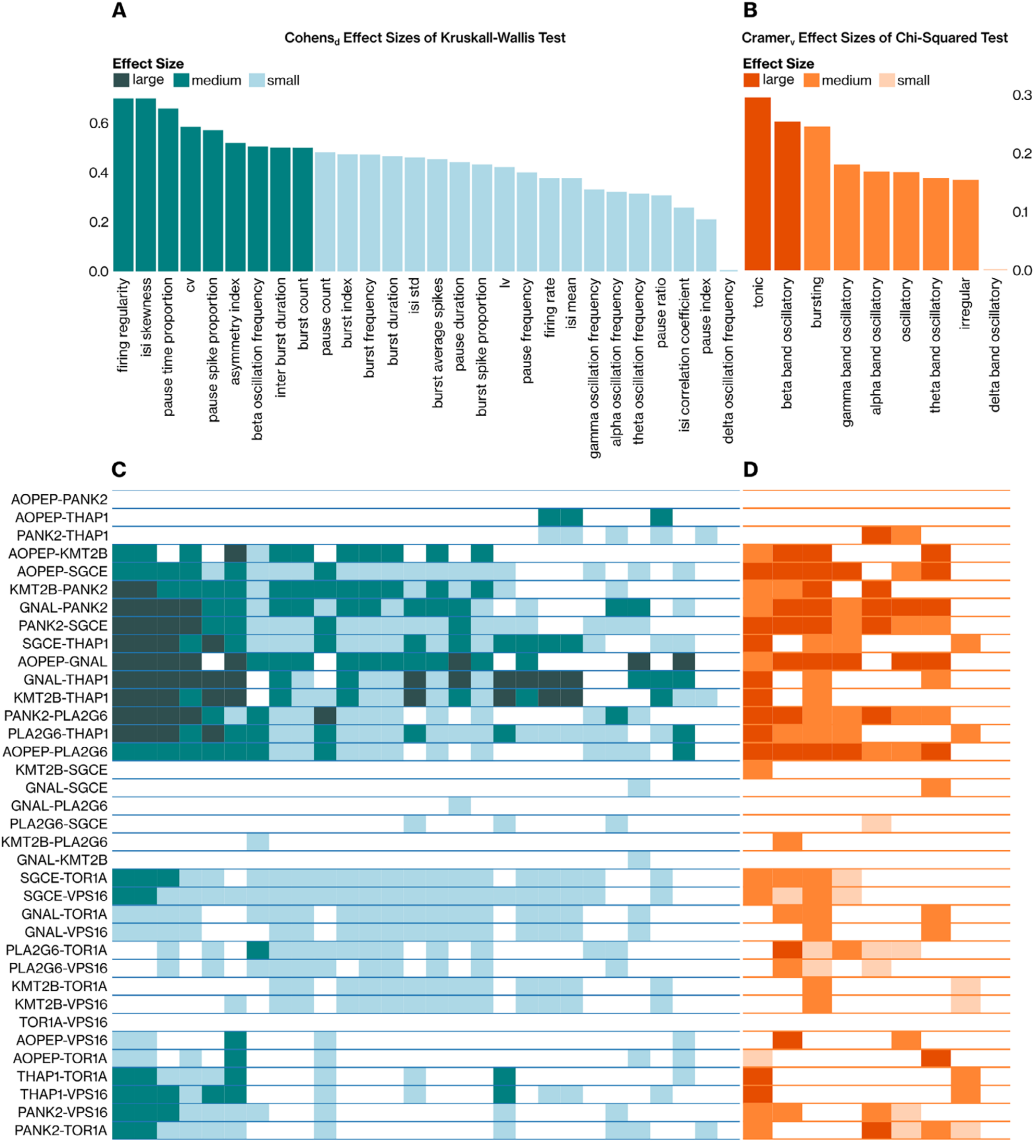
Dystonia genes demonstrate convergence in spiking regularity and neural bursts. The gene groups *AOPEP*, *PANK2*, and *THAP1*; *GNAL*, *PLA2G6*, *KMT2B*, and *SGCE*, and *TOR1A* and *VPS16* were formed based on the observed similarities in their pallidal neural patterns. Nonparametric statistical comparisons of neural features were performed with the Kruskal–Wallis (A) and chi‐square (B) tests. For post hoc analyses, we utilized the (C) Mann–Whitney *U* and (D) Fisher Exact tests. In all the statistical analyses, the *p* values were corrected with the Holm–Bonferroni method, and *p* ≤ 0.05 was considered to indicate statistical significance. The effect sizes were only reported when statistical significance was detected. The interpretation of the effect sizes used is denoted with color codes. [Color figure can be viewed at www.annalsofneurology.org]

All inspected neural features, besides delta band oscillations, showed statistically significant differences in at least one gene compared to the rest (*p* ≤ 0.05, Kruskal‐Wallis and chi‐square tests with Holm‐Bonferroni correction; Fig 2A, B). Four firing regularity, 2 neural burst and pause‐related features, and beta band oscillation frequency showed medium effect sizes. This indicated that differences in neural dynamics between dystonia genes were dominated by spiking regularity‐related observations. Comparisons of the presence of neural phenomena through the chi‐square test yielded similar outcomes, where the occurrence of tonic spiking and beta band oscillations showed large effect sizes (Cramer's v ≥ 0.25; see Fig 2B). To assess pairwise differences between the neural activity associated with these genes, we also performed ad hoc statistical tests (see the Methods section; Fig 2C, D). Refining the results of the previous section, the following groups of genes were defined based on similarity in overall pallidal dynamics: *AOPEP*, *PANK2*, and *THAP1*, and *GNAL*, *PLA2G6*, *KMT2B*, and *SGCE*; and finally, *TOR1A* and *VPS16* genes.

The most prominent differences were observed between the *PLA2G6*‐*THAP1* pair with 30 of 37 (81.08%) neural features displaying significant differences (*p* ≤ 0.05). Significant differences were also noted when *SGCE* was compared with *THAP1*, *VPS16*, *PANK2*, and *AOPEP* (> 70%; see Fig 2C). Firing regularity and interspike interval (ISI) skewness features showed significant differences in 22 gene pairs of 36 (61.11%). The majority of these significant differences (n = 18/22, 81.82%) observed for firing regularity and ISI skewness metrics had at least a medium effect size (Cohen's d ≥ 0.5), indicating once again the importance of spiking regularity‐related metrics. The value distribution of the delta band oscillation frequency metric was homogeneous across all genes (*p* > 0.05; see Fig 2C). The results from binary feature analysis were consistent with those from continuous feature analysis. Notably, *AOPEP* and *PANK2* neurons exhibited similar neural behaviors (bursts, oscillations, etc., Fisher's exact test, *p* > 0.05; see Fig 2D).

According to the OVR statistical comparison, 7 burst‐related neural features displayed differences across dystonia genes under consideration, highlighting neural bursts, along with spiking regularity, as another potential modulating factor (Supplementary Fig S5).

### Grouping Dystonia Genes Via Tonicity, Neural Bursts, and Oscillations

The results described above highlighted the relevant differences in neural features across dystonia genes and suggested the possibility of grouping the latter based on the former. We then proceeded with two stratification approaches to group dystonia genes (see the Methods section).

**FIGURE 3:**
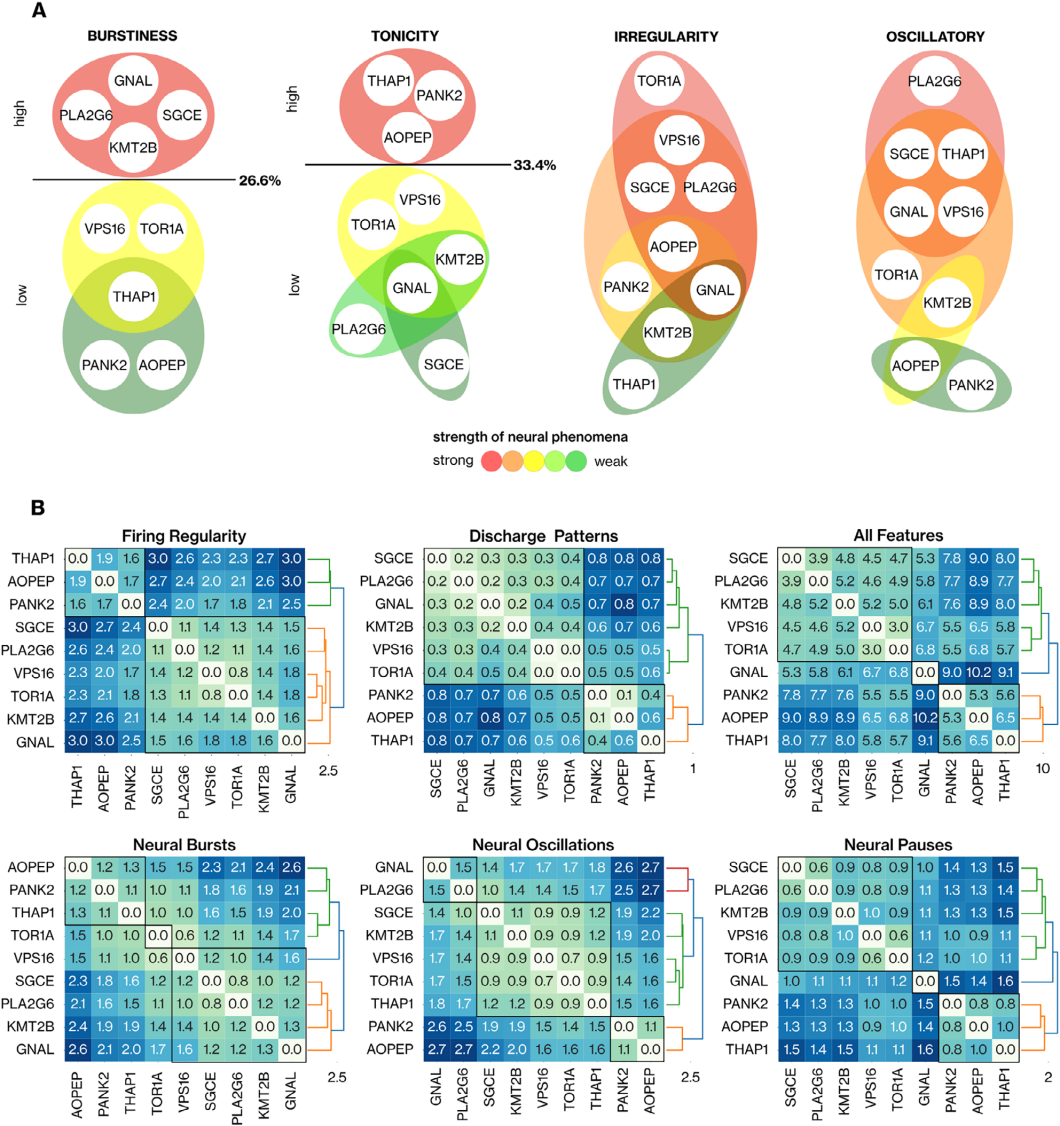
Stratification strategies yield consistent outcomes in the first‐order statistical comparison of neural features across dystonia genes. (A) Threshold‐based stratification for the fraction of neurons showing burstiness, tonicity, irregularity, and oscillatory behaviors. The threshold values were defined with the one‐sided Fisher's exact (*p* ≤ 0.05) test for low and high boundary genes (see the Methods section). (B) Cumulative distance matrices were computed with the Jensen–Shannon distance and hierarchical clustering approach using the unweighted pair group method with the arithmetic mean (UPGMA) algorithm to define clusters. The dendrograms indicate the agglomerative clustering of different genes for each neural dynamic. Distance‐based stratification is conducted for (*top*) firing regularity, discharge patterns, all neural features, (*bottom*) neural bursts, oscillations, and pauses. [Color figure can be viewed at www.annalsofneurology.org]

In the first approach, we aimed to establish statistically derived thresholds for the rate of neural phenomena to categorize gene groups. The fraction of bursting neurons partitioned the profiles into two distinct groups separated by a threshold of 26.6%. *GNAL*, *PLA2G6*, *SGCE*, and *KMT2B* neurons exhibited a significantly greater rate of burstiness (*p* ≤ 0.05, Fisher's Exact test with FDR correction) than the remaining genes. *PANK2* and *AOPEP* neurons showed significantly lower bursting activity than the other genes, except for *THAP1* (Fig 3A). Complementarily, *AOPEP*, *PANK2*, and *THAP1* demonstrated a significantly greater percentage of regular spiking neurons (tonicity ≥ 33.4%), with *SGCE* neurons displaying the least amount of tonic activity (see Fig 3A). The analysis of oscillatory behavior across all frequency bands revealed that *PANK2* and *AOPEP* neurons oscillate less (≤ 59.3%) than neurons of remaining dystonia‐causing genes. Consistent with burstiness, *PLA2G6* and *GNAL* neurons exhibited the highest level of oscillations (see Fig 3A). This analysis suggested the following characterization: *AOPEP* and *PANK2* neurons are highly regular and exhibit low levels of bursting and oscillatory behaviors. *GNAL*, *PLA2G6*, and *SGCE* neurons exhibit a high degree of burstiness, and prominent oscillations coupled with markedly low spiking regularity. Neurons of *TOR1A* and *VPS16* consistently demonstrated the highest degree of spiking irregularity.

In the second approach, we conducted an aggregated distance‐based partitioning of dystonia genes by comparing the distributions of neural features (see the Methods section; Fig 3B). The grouping based on the firing regularity and discharge patterns produced consistent results. *AOPEP*, *PANK2*, and *THAP1* formed one group, whereas the remaining genes constituted another group, aligning with the tonicity stratification observed in the previous section (see Fig 3B, top row). These outcomes were anticipated, as spiking regularity among *AOPEP*‐*PANK2*, *PANK2*‐*THAP1*, and *AOPEP*‐*THAP1* gene pairs were mostly comparable (see Fig 2C, D). Clustering by cumulative distance matrices of 8 bursting‐related features (see Fig 3B, bottom row) yielded consistent results with threshold‐based burstiness stratification (see Fig 3A).

The consistency between the two stratification strategies is noteworthy. Both the threshold and distance‐based categorizations of genetic dystonia syndromes for specific neural behaviors yielded matching results. This finding implies that rather than relying on complex neural features, we can gain insights into potential causative genes by simply examining the fraction of neurons exhibiting specific neural dynamics based on the threshold ranges we provided.

### Pallidal Firing Regularity Is Modulated by the Severity of Motor Symptoms

**FIGURE 4:**
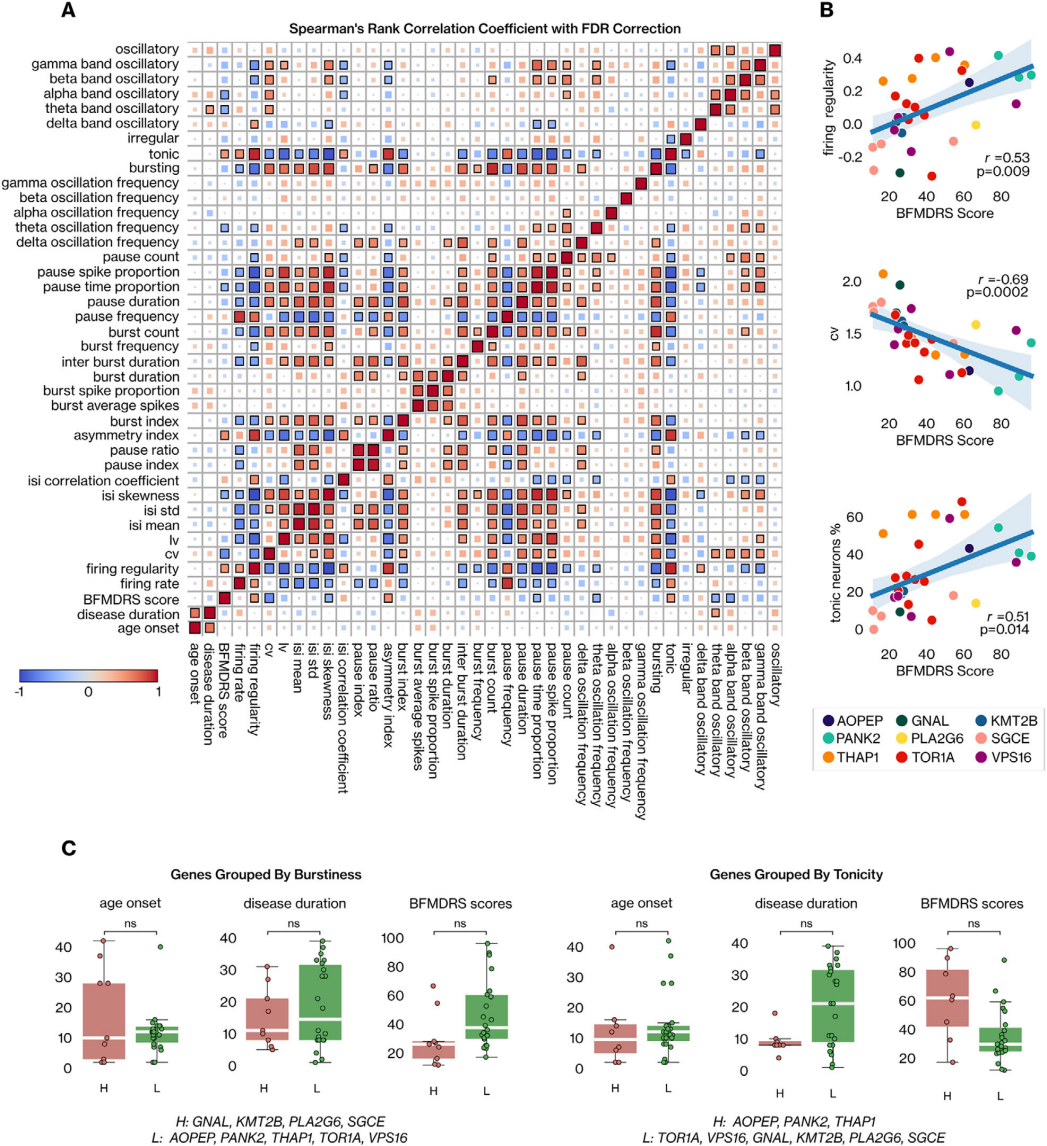
The relationship between neural features and the preoperative motor severity of dystonic symptoms. (A) Spearman's correlation coefficient with the permutation test is used to assess the statistical significance of the linear relationship between neural and clinical features. Benjamini/Hochberg (false discovery rate [FDR]) multiple comparison correction is applied to the *p* values of measured correlations. The magnitude of the correlation between two features is indicated by the size of the square, and significance is denoted by a black border around the corresponding square. (B) Significant linear correlations between firing regularity, coefficient of variation (CV), the rate of tonic neuron metrics, and preoperative Burke Fahn Marsden Dystonia Rating Scale (BFMDRS) score are illustrated with a line plot. The scatter plot is coded based on the genetic etiology of each patient. (C) The patient groups are generated based on regularity (right) and burstiness (left) grouping (see Figure 3A). Age of onset, disease duration, and preoperative BFMDRS scores are compared between patient groups using the Mann–Whitney *U* test with Holm‐Bonferroni correction. [Color figure can be viewed at www.annalsofneurology.org]

In Parkinson's disease, motor symptom severity correlates with the beta‐power of subthalamic local field potentials (LFPs)^37^ and the spiking properties of basal ganglia nuclei.^38^ To assess the influence of motor symptom severity, age of onset, and disease duration on globus pallidus neural activity, independent of genetic factors, we measured Spearman's rank correlation between these variables and the mean values of neural metrics per patient. We conducted a permutation test to assess the significance of the correlation, employing FDR correction for multiple comparisons. Finally, we compared these three variables within our patient groups according to the clustering achieved for firing regularity and neural bursts (Fig 4).

The total Burke Fahn Marsden Dystonia Rating Scale (BFMDRS) score exhibited a significant correlation with firing regularity metrics, as did the proportion of tonic, theta‐band oscillatory, and alpha‐band oscillatory neurons (see Fig 4A). Specifically, an elevated BFMDRS score strongly indicates a decreased CV and an increased fraction of tonic neurons in dystonic patients (see Fig 4B). From this perspective, the severity of dystonia initially appeared to be a potential confounding factor in our results related to regularity‐based genetic profiling. However, upon comparing the total BFMDRS scores among patient profiles defined by firing regularity metrics (see Fig 4B), we did not observe a significant difference (*p* > 0.05, Mann–Whitney *U* test with Holm‐Bonferroni correction; see Fig 4C). These findings suggest that while there is a significant relationship between dystonia severity and neural activity in patients with dystonia, it does not significantly affect bursting and regularity‐based genetic profiling.

### Neural Decoding of Dystonia Genes

**FIGURE 5:**
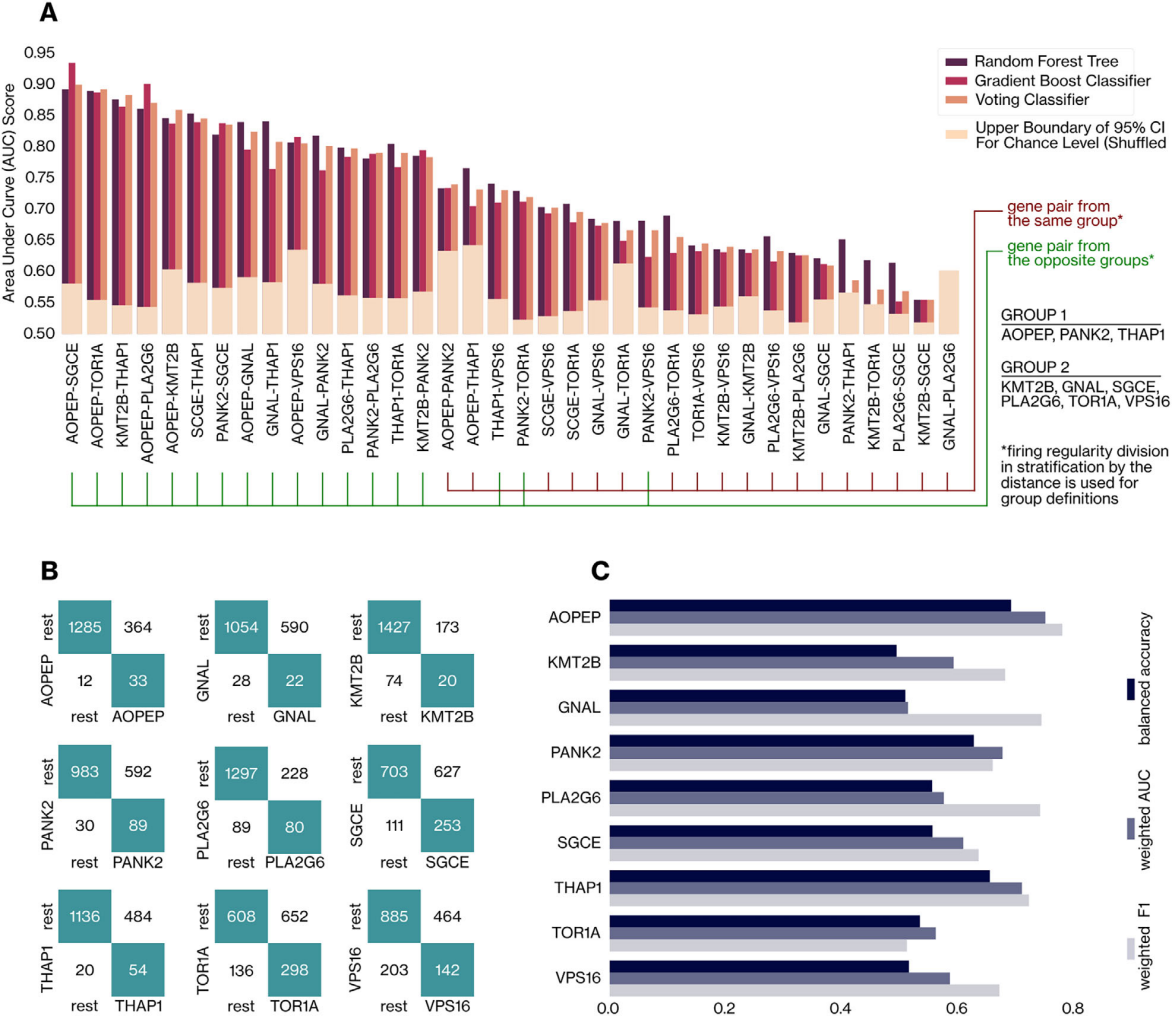
Effect of firing regularity on the classification of dystonia genes on neural decoders. (A) The weighted area under the curve (AUC) scores (averaged across 5 folds during stratified cross‐validation) of a random forest tree, gradient boost, and voting classifiers are given for 36 pairs of dystonia genes. Model validation was conducted through a 100‐iteration bootstrap test, in which randomness was introduced by shuffling gene information across neurons and training new models in each iteration. The 95th percentile of the resulting random‐event performance distribution represents the upper threshold for random classification performance (Supplementary Section 5). (B) The confusion matrices of one‐versus‐rest (OVR) classification were constructed with random forest tree models. (C) The balanced accuracy, the weighted AUC and F1 scores of OVR classifiers of dystonia genes are presented with separate color coding. [Color figure can be viewed at www.annalsofneurology.org]

Apart from statistical characterization, we investigated the potential of machine learning approaches to discriminate neural signatures of dystonia genes. Accordingly, we first tested the performance of machine learning models (see the Methods section) based on neural features in discriminating gene pairs. Optimal results were achieved by discriminating *AOPEP* from *PLA2G6*, *GNAL*, and *KMT2B*. The model constructed for *GNAL* and *PLA2G6* neurons failed to surpass the performance expected by random chance (Fig 5A, Supplementary Table S8). Upon interpreting the findings, a notable pattern emerged regarding high‐ and poor‐performing OVO models. High‐performing models were trained using neurons from genes belonging to opposite groups where we considered group definitions of two stratifications for firing regularity (see Fig 3A, B, top rows).

We then tested the performance of the assemblers in identifying single genes. The *AOPEP*, *PANK2*, and *THAP1* genes exhibited high distinguishability from other genetic dystonia syndromes. Meanwhile, *GNAL* and *KMT2B* neurons performed relatively poorly (see Fig 5B, C, and Supplementary Table S9).

## Discussion

Our study revealed shared and diverse pallidal patterns among dystonia genes by analyzing intraoperative MERs. Spiking regularity and neural bursts were the two main neural dynamics to discern pallidal activity across genetic dystonia syndromes. We identified commonalities regarding the tendency of *GNAL*, *PLA2G6*, *SGCE*, and *KMT2B* neurons to burst and oscillate more; meanwhile, *AOPEP*, *PANK2*, and *THAP1* neurons exhibited prominent spiking regularity. Notably, we demonstrated that dystonia genes sharing a common molecular pathway, such as *PLA2G6* and *PANK2*, can manifest opposite neural behaviors in the globus pallidus.

### Pallidal Neural Activity for Genetic Dystonia Syndromes

The neural activities of dystonia genes in the globus pallidus are still largely unknown. To our knowledge, no study investigated the pallidal electrophysiology for *AOPEP*, *KMT2B*, *PLA2G6*, *SGCE*, and *VPS16* genes. We recently presented neural activity in the globus pallidus for *GNAL* ^39^ and *PANK2* ^40^ cases for the first time. In *TOR1A*, a recent study reported a firing rate of 52.2 spikes/s for pallidal neurons under local anesthesia (LA), with 53% of GPe neurons and 58% of GPi neurons exhibiting bursts and tonic neurons constituting approximately 33% of pallidal neurons.^13^ The observed difference in overall burstiness between our and this study can be attributed to variations in bursting and tonic neuron definitions and the influence of anesthetic agents.

Two other patients with *TOR1A* reported a firing rate of approximately 26 spikes/s under propofol general anesthesia (GA).^12^, ^41^ Two comparative studies examined pallidal patterns in *THAP1* and *TOR1A* pathogenic variant carriers.^10^, ^11^ The mean firing rate, burst index, and CV scores were similar between the two groups under both LA^11^ and GA combined with propofol and remifentanil,^10^ consistent with our results. The reported proportions of bursting, tonic, and irregular neurons for *THAP1* were in line with our study.^11^ Zittel et al also reported similar firing rates for both *THAP1* and *TOR1A*, which were similar to our results under propofol anesthesia.^10^

### Interaction between Genetic Etiology and Dystonia Circuitry

Previously, common electrophysiological patterns among dystonia genes were suggested to indicate a shared mechanism at the level of neural circuits.^22^ The differences in varying responses to GPi‐DBS among monogenic dystonia cases were implicated to indicate potential differences in the underlying network dysfunction.^42^, ^43^ In this regard, our findings may hint at possible convergence among dystonia genes at the neural circuitry level based on their pallidal neural signatures and further support the convergence paradigm previously demonstrated at molecular, cellular, and regional levels in the brain.

### Potential Impact of Genetic Etiology on DBS Effectiveness

Our work has potentially strong clinical implications, as it may explain a possible mechanism accounting for the variability of DBS efficacy across genetic profiles. Although the exact mechanism of DBS is surely multifaceted and not completely known, there is a vast consensus on the fact that the mechanisms involve the desynchronization of neuronal population and hence the suppression or at least the reduction of pathological oscillations.^44^, ^45^ This is valid for beta oscillations in Parkinson's Disease^46^ and low‐frequency oscillations of 4 to 12 Hz in dystonia.^47^, ^48^ Here, we have identified two well‐defined clusters of dynamics: genetic profiles leading to strong bursts and low tonicity and genetic profiles with weak bursts and high tonicity (see Fig 3A, B). Desynchronization mechanisms can only impact the genes in the first group, as the second group lacks strong overall synchronization in the first place. Crucially, the genetic profiles in which DBS is known to be effective (*TOR1A*,^4^, ^49^, ^50^ *GNAL*,^4^ *KMT2B*,^4^ and *SGCE* ^4^) all belong to the first group. Instead, *THAP1*, the genetic profile for which DBS is known to be less effective,^4^, ^49^, ^50^ belongs to the second group and is associated with the most prominent spiking regularity (see Figs 1B and 3A). The other two profiles where DBS is expected to be less effective are *PANK2* and *AOPEP*, with no strong consensus on DBS efficacy for *PANK2*,^4^, ^49^ and no conclusive studies for *AOPEP*. Combining the clinical evidence with our neurophysiological observations suggests that variability in DBS effectiveness across genetic profiles may stem from differences in whether genes are associated with bursting or tonic pallidal activity. We also confirmed this by comparing the change in BFMDRS scores from preoperative to 1‐year postoperative evaluations between these 2 groups in our cohort (see Table 2). Overall, patients with pathogenic mutations in genes associated with tonic pallidal activity (*AOPEP*, *THAP1*, and *PANK2*) showed significantly lower responses to GPi‐DBS compared with the remaining patients, with decreases in BFMDRS scores of −13.6% and −51.6%, respectively (*p* = 0.019, one‐sided Mann–Whitney *U* test).

For genetic profiles associated with strong tonicity, there may be insufficient abnormal synchronization in the GPi, making GPi‐DBS potentially less effective. Possible solutions could include selecting different targets (although synchronization patterns are often shared among basal ganglia nuclei) or designing specific stimulation patterns tailored to these profiles.

### Implications for Adaptive DBS Treatment

Previous functional MRI (fMRI) studies have observed abnormal activity in the cerebello‐striato‐cortical network in dystonia patients, even at rest.^42^ Here, we stratified the degree of pallidal spiking regularity and bursts across dystonia genes into two groups at the resting state. The distinct bursting behavior across dystonia genes is particularly significant. Recently, periodic single‐neuron bursts were found to encode pathophysiological subthalamic beta LFP oscillations in patients with Parkinson's disease.^51^ The authors demonstrated significant time‐frequency and phase‐coupling connections between single‐unit bursts and LFP activity, finding that spiking activity occurring outside of bursts showed no correlation with LFP. From this perspective, our discovery of potential varying effects of genetic etiology on single‐unit bursting behavior may indicate differing LFP activity across dystonia genes in the globus pallidum.

GPi‐DBS was demonstrated to improve dystonia by restoring more physiological firing patterns in the globus pallidum.^47^, ^52^ Research emphasizes the importance of low‐frequency activity in the dystonic GPi, suggesting its potential as a biomarker for closed‐loop DBS in dystonia. Therefore, studying the effect of genetic etiology on the low‐frequency component of pallidal electrophysiology may lead to personalized adaptive DBS treatments tailored to the underlying genetic causes of dystonia in patients.

### Converging Biological Pathways in Dystonia Genes

In our work, we focused solely on investigating converging pallidal neural patterns among dystonia genes and their implications for DBS treatment, without addressing the connection between molecular mechanisms of these genes with symptomogenesis, as this is beyond the scope of our paper (see Limitations). In this subsection, we summarized all converging biological pathways reported across different anatomic scales.

At the molecular level, dysfunctions of these genes have distinct mechanisms and can be categorized as follows: alterations in protein turnover and post‐translational modifications (*AOPEP*), mitochondrial and energy homeostasis dysfunction (*PANK2* and *PLA2G6*), autophagy (*VPS16*), perturbation of gene transcription during neurodevelopment (*KMT2B* and *THAP1*), alteration in intra‐ and extracellular structural elements (*SGCE*), disruptions in intracellular transport mechanisms (*TOR1A*), and altered dopamine signaling (*GNAL*).^18^, ^53^

These genes have complex interactions that regulate essential cell functions. *THAP1* is implicated in the transcriptional regulation of *TOR1A*, and both of these genes were shown to impact the expression of proteins associated with the eIF2α pathway.^54^, ^55^ Mutated *KMT2B* fibroblasts exhibit reduced *TOR1A* and *THAP1* mRNA and protein expression, suggesting that KMT2B functions as an upstream regulator of other genetic dystonia syndromes.^56^ Mutations in *GNAL* are linked to defects in dopamine receptors, attributed to its role in cAMP metabolism in striatal neurons.^57^ *TOR1A*, *SGCE*, and *THAP1* mutations were also demonstrated to impact striatal dopaminergic signaling in animal models.^58^, ^59^, ^60^ Reduced D2 receptor expression is found for *THAP1*, *TOR1A*, and *KMT2B*.^56^, ^61^ In this context, aberrant dopamine signaling serves as a downstream effect of unrelated genetic defects.^18^ Concerning the functional metabolism of the globus pallidus, a recent study indicated that individuals with mutations in *KMT2B*, *THAP1*, and *SGCE* exhibited hypoactivity, contrasting with that of patients with *PANK2* and *TOR1A*. Moreover, putamen hypometabolism was a common sign of pathogenic *TOR1A*, *SGCE*, *THAP1*, and *KMT2B* mutations.^62^ Radiologically, pallidal hypointensity was observed in individuals with *PANK2*,^63^ *PLA2G6*,^64^ *THAP1*,^65^ *VPS16*,^66^ or *KMT2B* ^56^ mutations.

**FIGURE 6:**
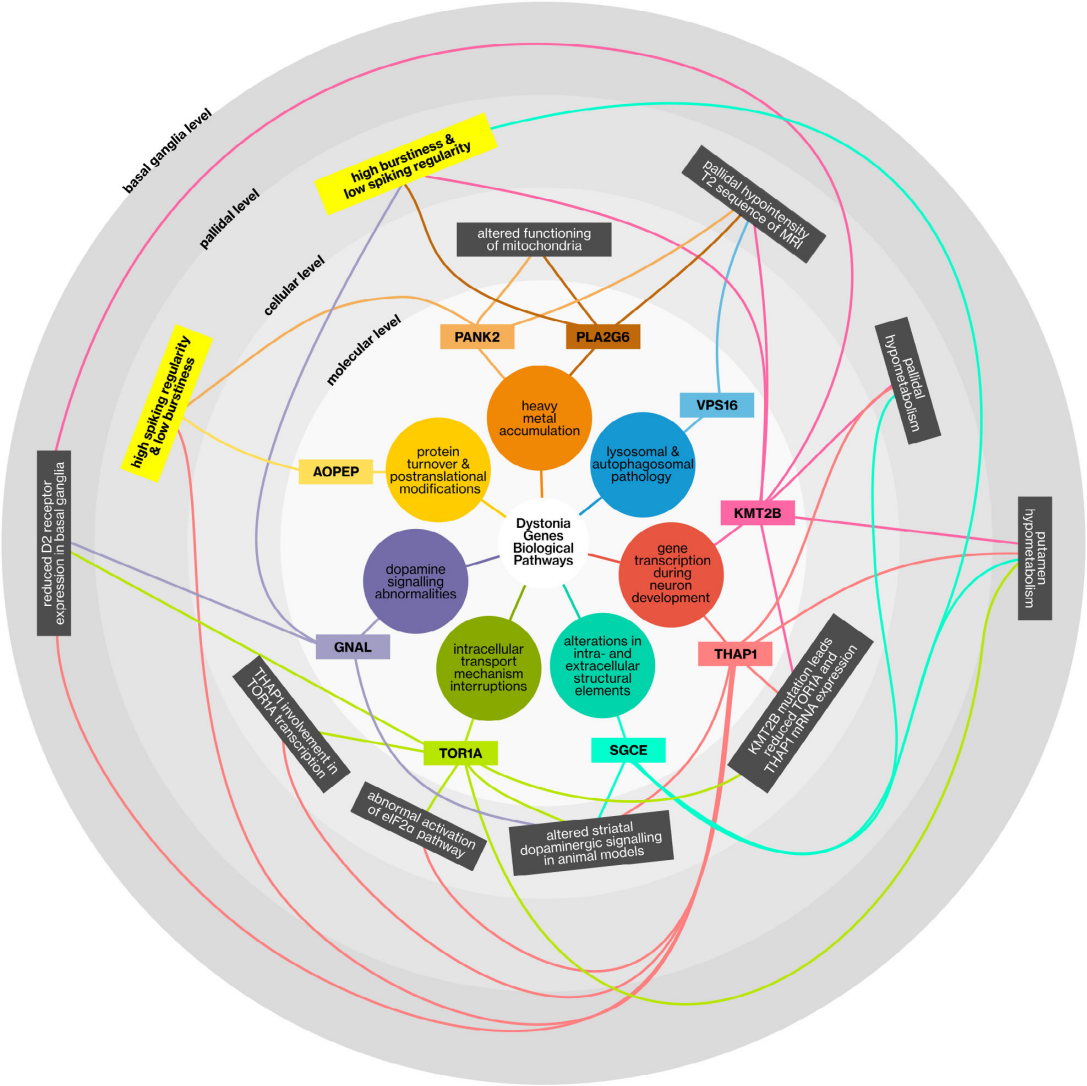
The convergent phenomenon observed between dystonia genes at different anatomical levels. The previous literature findings regarding all the convergent phenomena observed between the *AOPEP*, *KMT2B*, *GNAL*, *PANK2*, *PLA2G6*, *SGCE*, *THAP1*, *TOR1A*, and *VPS16* genes at the molecular, cellular, pallidal, and basal ganglia levels are presented. The anatomic levels were ordered from inner to outer circles according to their sizes in a hierarchical manner, going from dynamics occurring at the level of the whole basal ganglia (outermost) to biological pathways at the molecular level (innermost). Outer levels contain anatomic structures in inner levels. Each convergent phenomenon within a specific level is depicted with a dark gray rectangle (reported in the literature), and genes converging into this phenomenon are linked with color‐coded lines. The two new convergent phenomena that we observed in our study are indicated by yellow rectangles at the pallidal level. [Color figure can be viewed at www.annalsofneurology.org]

We illustrated all convergent biological phenomena presented in previous works at different anatomic scales, along with our pallidal electrophysiological findings in Figure 6.

### Limitations

A limitation inherent to our study is the relatively constrained size of our patient cohort. This stems from the rarity of genetic dystonia syndromes. A larger patient cohort would undoubtedly improve the robustness and generalizability of our findings. However, we systematically used a range of statistical tests and analyses to ensure validation and coherence among the different analyses.

Establishing a direct link between the biological functions of studied genes and the neural pattern they presented is challenging with our data and beyond the scope of our study. Factors such as the varying degrees of gene expression in the globus pallidum (*KMT2B* and *THAP1* being predominantly expressed in the cerebellum^67^), the crosstalk between basal ganglia and cerebellum^68^ complicate the ability to draw such conclusions. The crosstalk indicates that the cerebellum may have a direct impact on basal ganglia functions through connections between the dentate nucleus and both the subthalamic nucleus and the pallidum, highlighting reciprocal connections between the striato‐pallido‐thalamo‐cortical and cerebello‐thalamo‐cortical circuits.^68^, ^69^ Functional MRI studies further revealed concurrent impairments in the striato‐pallido‐thalamo‐cortical and cerebello‐thalamo‐cortical circuits.^70^, ^71^ Additionally, the existence of non‐manifesting carriers (NMCs) of the same genetic factor who do not present dystonic symptoms should be considered.^72^ Therefore, our observations regarding the role of dystonia genes on converging neural patterns are correlational and exploratory.

We did not find robust evidence supporting a connection between the type of inheritance patterns that dystonia genes follow and the convergent neural dynamics we observed. *AOPEP*, *PANK2*, and *PLA2G6* mutations exhibit autosomal recessive patterns (Supplementary Table S1). Although *AOPEP* and *PANK2* neurons largely exhibited similar neural behaviors in the globus pallidus, they consistently and significantly contrasted with *PLA2G6* neurons (see Figs 2 and 3). However, our observation regarding the influence of inheritance patterns on pallidal neural activity should be approached with caution, given that we had only one patient each for *AOPEP* and *PLA2G6*.

The administration of anesthetics during MER acquisition has been shown to introduce distortions and suppress neural activity within the basal ganglia in movement disorders.^12^, ^73^, ^74^, ^75^ The increased occurrence of quiet zones in the GPi and GPe due to anesthetic agents and the challenge in distinguishing high‐frequency discharge cells in the GPi from border cells^75^ influenced our decision to analyze GPi and GPe neurons together to avoid potential interferences. Propofol has dose‐dependent effects on pallidal neurons, resulting in a decrease in neural firing and an increase in burstiness, particularly in the GPi.^12^, ^73^, ^75^ The impact of remifentanil on pallidal activity in dystonic patients has not been determined, yet MER recordings taken after a combination of low‐dose propofol infusion and remifentanil were found to be most robust for GA.^75^ In this regard, our neurophysiological characterizations of dystonia‐causing genes are influenced by both propofol and remifentanil, but their uniform administration ensures comparable effects on MERs for all patients.

For most patients (23/31), we utilized 2 to 5 MER trajectories (central, anterior, posterior, lateral, and medial). Therefore, we hypothesize that these trajectories collectively cover a substantial portion of the GPi. However, we did not analyze how the position of neurons affects their activity. Although current literature lacks substantial evidence on spatial effects in GPi electrophysiology, this omission may introduce additional bias.

### Perspectives

Factors such as the age of onset, body distribution of symptoms, clinical expression of phenotype,^23^ and the presence of non‐motor symptoms can significantly vary between carriers of the same dystonia gene. On the other hand, genes with different molecular bases can converge into similar biological pathways for dystonia pathogenesis. Future comparative studies examining similarities and differences in pallidal and subthalamic LFP activity across dystonia genes could lead to optimized adaptive DBS (aDBS) treatment paradigms for patients with dystonia.

## Author Contributions

A.K., F.C., M.A., Z.I., D.A., J.C., M.C., H.P., M.Z., H.B., A.M., and L.M.R. contributed to the conception and design of the study. A.K., F.C., N.G.A., S.R., R.T., V.L., G.Z., B.G., R.E., L.M.R., and A.M. contributed to the acquisition and analysis of data. A.K., F.C., A.M., and L.M.R. contributed to drafting the text or preparing the figures.

## Potential Conflicts of Interest

Nothing to report.

## Supporting information


**Data S1.** Supporting Information.

## Data Availability

The processed MER data supporting the findings of this study are openly available in Zenodo at https://doi.org/10.5281/zenodo.13269430 in a tabular and fully anonymized format. The complete code for neural data processing, descriptive and predictive analyses, and plotting is available online on the GitHub repository: github.com/ahmetofficial/Genetic-DYT-Pallidal-Neural-Pattern-Analysis.
