# Supplementary material for: Spiking Patterns in the Globus Pallidus Highlight Convergent Neural Dynamics across Diverse Genetic Dystonia Syndromes
Source: Ann Neurol. 2025 Jan 30;97(5):826–44. doi: 10.1002/ana.27185 (PMC12010065; doi:10.1002/ana.27185)
Supplement: Supplementary file 1 — Data S1. Supporting Information. [file ANA-97-826-s001.docx]

**Supplementary Table 1. Clinical characteristics of genetic dystonia syndromes reported in the literature.**

| **Dystonia**  **Gene** | **Clinical Characteristics** |
| --- | --- |
| ***AOPEP*** | It is a form of isolated dystonia with recessive inheritance^1^ and emerges in childhood to early adulthood initially affecting hands^2–5^ or lower limbs^3^. Postural and movement abnormalities spread to limbs and neck during the progression phase, with occasional articulation difficulties .^2–4^ Dystonia tends to be generalized^2–6^ sometimes with Parkinsonian features ^2,3,6^. Cognitive issues are typically not prominent.^2–6^ |
| ***GNAL*** | *GNAL* mutations have a dominant inheritance pattern with 94% penetrance.^7–9^ The majority of *GNAL* patients experience cervical dystonia, approximately 84%, with an average onset age of 35.4 years.^10–17^ The symptoms generally remain focal in the neck area or multifocal (cranial or upper limb involvement) (~52% and ~36%, respectively) without significant cognitive and psychiatric issues.^18,19^ |
| ***KMT2B*** | *KMT2B* mutations cause autosomal dominant dystonia that usually emerges in childhood,^20–25^ primarily in the lower limbs^26–28^. It becomes generalized (~83%)^25^ with prominent oromandibular, cervicocranial, and laryngeal involvement.^20–25,27,29–31^ Patients can experience mild to moderate cognitive impairment (~51%), and varying degrees of facial dysmorphia or short stature (~60%).^23–25^ |
| ***PANK2*** | Traditional classifications of *PANK2* mutations include the early-onset, quickly progressing "classic" form and the later-onset, slower-progressing "atypical" form with recessive inheritance pattern.^32–37^ Regardless, complex dystonia is present in almost all patients (~97%), along with common symptoms like dysarthria (~83.2%), oromandibular dystonia (~80%), and gait issues (~55.1%). ^33,35,37–45^ Approximately 43.3% and 42% of all patients additionally experience cognitive and psychological/behavioral problems, respectively.^37^ |
| ***PLA2G6*** | *PLA2G6*-associated neurodegeneration leads to rapidly progressing, autosomal recessive dystonia-parkinsonism.^46–48^ At the onset, gait issues and postural instabilities are widely reported.^49–56^ The clinical condition gradually deteriorates leading to dystonia, severe spasticity, and cognitive dysfunction. Neuropsychiatric symptoms, including depression, psychosis, and cognitive decline, are common.^46,49–53^ |
| ***SGCE*** | In *SGCE* carriers*,* mutations are inherited in an autosomal-dominant pattern and it is characterized by myoclonic jerks, usually the most and main disabling feature, predominance in the neck and upper limbs.^57–64^ Initial symptoms are restricted to the neck and upper extremities and remain mostly focal or segmental,^65–67^ Patients may experience psychiatric issues.^57,59,65,68^ |
| ***THAP1*** | *THAP1*-associated *dystonia* is an autosomal-dominant, adolescent-onset dystonia ^69–71^ with broad clinical phenotype^71,72^. Onset symptoms generally involve upper limbs(~33%), neck(~27%), or craniofacial/laryngeal(~25%) muscles, while lower limb involvement is infrequent.^69–71,73,74^ The primary disability for patients stems from craniocervical involvement.^72,75,76^ Particularly, laryngeal dystonia is prevalent and associated with several speech difficulties.^69,72,76^ |
| ***TOR1A*** | *TOR1A* is the most common and studied dystonia gene with autosomal-dominant inheritance.^77,78^ Characteristically, it initially affects the limbs(~60%) as focal dystonia with an early onset.^71,75^ Motor symptoms tend to be generalized while craniofacial or laryngeal involvement is rare.^71,79,80^ Nonmotor symptoms are generally not reported (>90% missing).^71^ |
| ***VPS16*** | Variants in *VPS16* cause autosomal-dominant isolated dystonia with early onset^1^ with initial manifestations mainly cervical dystonia^81–83^ and writer’s cramp^81,84–90^. In VPS16 carriers, symptoms vary, with most progressing to generalized dystonia and approximately 20% maintaining focal or multifocal symptoms.^1^ Additionally, intellectual disabilities and neuropsychiatric conditions can emerge for patients (~20% and ~30%, respectively).^88,89^ |

**Supplementary Table 2. Clinical profile of the studied population.**

| Patient ID | Gene | Clinical Phenotype | Familiarity/Segregation Analysis | Patient Reference |
| --- | --- | --- | --- | --- |
| Pat1 | *VPS16* | Generalized tremulous dystonia with prominent laryngeal, oromandibular, and appendicular involvement (right>left); writer’s cramp | Negative.  Not available segregation analysis | Monfrini et al.^91^  (Patient E) |
| Pat2 | *VPS16* | Speech-induced oromandibular dystonia, right upper limb jerky dystonia, and writer’s cramp; mild right torticollis | Negative.  Not available segregation analysis | Monfrini et al.^91^  (Patient F) |
| Pat3 | *SGCE* | Generalized myoclonic-dystonia, mainly involving the cranial-cervical region and upper limb | Negative.  Not available segregation analysis | Not yet described |
| Pat4 | *VPS16* | Generalized tremulous dystonia with severe jerky movement (left torticollis, right upper limb striatal toe); voice tremor; writer’s cramp with writing tremor | Maternal inheritance (Mother with generalized dystonia and parkinsonism; sibling Pat6) | Monfrini et al.^91^  (patient C.II.1) |
| Pat5 | *VPS16* | Cranial-cervical involvement with dysphonia, dystonic dysarthria, and writer’s cramp | Maternal inheritance (Mother with generalized dystonia and parkinsonism; sibling Pat5) | Monfrini et al.^91^  (patient C.II.2) |
| Pat6 | *AOPEP* | Generalized dystonia with axial parkinsonism | Maternal inheritance (Mother healthy heterozygosis carrier) | Garavaglia et al.^92^  (Patient III.6 family 2) |
| Pat7 | *SGCE* | Myoclonus dystonia with axial distribution, mainly proximal upper limb and head | Negative.  Not available segregation analysis | First described by  Hess et al.^93^ |
| Pat8 | *THAP1* | Generalized dystonia, with prominent cranial-cervical dystonia and dysarthria | Not available | Danielsson et al.^94^  (Patient #8) |
| Pat9 | *THAP1* | Generalized dystonia with mainly trunk and right upper limb involvement | Not available | Danielsson et al.^94^  (Patient #4) |
| Pat10 | *THAP1* | Generalized dystonia with cervical, trunk, and right upper limb involvement | Not available | Danielsson et al.^94^  (Patient #5) |
| Pat11 | *THAP1* | Cervical dystonia and dysarthria | Negative, mother analysis wild type | Danielsson et al.^94^  (Patient #6) |
| Pat12 | *PANK2* | Generalized dystonia with mainly cervical and axial involvement | Negative.  Not available segregation analysis | Romito et al.^95^ |
| Pat13 | *KMT2B* | Generalized dystonia with severe trunk and cervical involvement; dysarthria | Negative.  Not available segregation analysis | Not yet described |
| Pat14 | *TOR1A* | Generalized dystonia with mainly trunk and lower limb involvement | Negative.  Parents’ segregation analysis wild type | Not yet described |
| Pat15 | *TOR1A* | Generalized dystonia with severe tortipelvis and lower limb dystonia | Negative.  Not available segregation analysis | Not yet described |
| Pat16 | *TOR1A* | Generalized dystonia with mainly trunk and lower limb involvement | Negative.  Parents’ segregation analysis wild type | Duga et al.^96^  (Patient #12) |
| Pat17 | *TOR1A* | Generalized dystonia with mainly trunk and upper limb involvement | Negative.  Not available segregation analysis | Not yet described |
| Pat18 | *TOR1A* | Generalized dystonia with mainly cervical, trunk, and lower limb involvement | Not available segregation analysis | Not yet described |
| Pat19 | *TOR1A* | Generalized dystonia with mainly trunk and lower limb involvement | Negative.  Not available segregation analysis | Not yet described |
| Pat20 | *TOR1A* | Generalized dystonia with mainly trunk and upper limb involvement | Not available segregation analysis.  Son is affected by generalized dystonia due to the same mutation | Not yet described |
| Pat21 | *TOR1A* | Segmental tremulous dystonia with severe cervical and upper limb involvement | Negative.  Not available segregation analysis | Not yet described |
| Pat22 | *TOR1A* | Generalized dystonia with mainly trunk and lower limb involvement | Negative.  Not available segregation analysis | Not yet described |
| Pat23 | *KMT2B* | Generalized dystonia with mainly trunk, upper limbs, and oromandibular involvement | Negative.  Parents’ segregation analysis wild type | Carecchio et al.^97^  (Patient #6) |
| Pat24 | *SGCE* | Myoclonus dystonia with cervical and upper limb distribution | Father and sister with myoclonus dystonia | Not yet described |
| Pat25 | *SGCE* | Myoclonus dystonia with cervical and upper limb distribution | Negative.  Not available segregation analysis | Lunardini et al.^98^ |
| Pat26 | *GNAL* | Cranio-cervical tremulous dystonia | Negative.  Not available segregation analysis | Romito et al.^99^ |
| Pat27 | *GNAL* | Cranio-cervical tremulous dystonia, with laryngeal involvement | Mother and two brothers with GNAL mutation and mainly cervical dystonia phenotype | Carecchio et al.^100^  (Patient III:4) |
| Pat28 | *PANK2* | Generalized dystonia with severe dysarthria | Negative.  Not available segregation analysis | Not yet described |
| Pat29 | *PANK2* | Generalized dystonia with severe trunk and lower limb involvement; gait disorder | Negative.  Parents’ segregation analysis wild type | Not yet described |
| Pat30 | *VPS16* | Generalized dystonia with severe axial involvement; severe dysarthria | Positive.  Father with the same mutation, as phenotype rest and postural jerky tremor, upper limb akinesia. | Not yet described |
| Pat31 | *PLA2G6* | Generalized dystonia with severe trunk, cervical, and lower limb involvement | Negative.  Parents’ segregation analysis wild type | Not yet described |

# Electrophysiological Data Preprocessing

## Spike Sorting

In principle, spikes fired by a neuron recorded by a given electrode have a distinct shape that depends on the morphology of the corresponding dendritic tree and the distance and orientation relative to the recording site, among other factors.^102^ Spike detection and sorting were conducted using the MATLAB toolbox Wave_Clus which is an unsupervised spike-sorting algorithm that uses wavelet decomposition to extract features of spike waveforms and superparamagnetic clustering (SPC) to cluster spikes into single neural activities.^103^ Briefly, we detected multiunit activity in raw recordings by employing a double-threshold-based approach in which the data points exceeded the 4th time the standard deviation of the recording compared to baseline activity, the mean, was considered multiunit activity. From these multiunit events, the toolbox measures 64 Haar wavelet coefficients to characterize the spike shapes at different scales and times. The SPC approach was subsequently used to discriminate the spiking activity of individual neurons. We employed a relatively conservative threshold (4th time the std from baseline) to reduce the effects of noise components in the operating room on single-unit events, and we uniformly applied this threshold across MERs of all patients in our cohort.

## Single-Unit Activity Definition

The single-unit activity (SUA) represents the section of the raw data that belongs to the spiking of a single neuron. The [-0.5 +2.5] ms time interval around the peak values of all spikes was extracted from the raw data and stored as the neural activity of a single neuron. In the literature, single-unit activity refers to the separable single-unit spike train (SU-ST), not the non-separable multiunit spike train (MU-ST).^104^ MU-STs are formed by spikes from many neurons and have shapes that cannot be separated because of a low signal-to-noise ratio. The neurons contributing to the multiunit activity are relatively close to the electrode (for their spikes to be detected) but not close enough to enable the clustering of their shapes.^105^

## Stability criterion for sorted neurons

The stability of the detected neurons was investigated before proceeding with further analysis. This stability check of neurons was performed to avoid adding neurons to the dataset that did not have predicted neural activity or spike morphology. The adopted stability criterion^106^ checks four different aspects of sorted neurons. **(a)** More than 90% of the total area of the amplitude histogram had to be above the detection threshold. We suggest the temporal region in the raw recording, which shows distinguishable changes compared to the baseline to indicate spiking activity. For each spike, all the spiking peaks were compared with the defined double threshold. The next criterion was to determine whether 90% of the detected spike peaks were above this threshold level. **(b)** The mean waveform of the clustered activity of a neuron needs to have a typical action potential shape with a prominent peak. This step is completed via visual inspection during spike sorting. **(c)** The percentage of spikes occurring within 3 ms of each other had to be less than 1%. This criterion ensures that we do not involve a spiking activity that occurs in the refractory period. **(d)** The number of spikes detected was more than 20. For meaningful statistical conclusions, this threshold is defined to select the neurons. This stability criterion was applied to all the isolated neurons. Only the neurons that passed the stability criterion were accepted for the next steps.

**Supplementary Table 3. Single-Unit Activity Distribution Across Genetic Variants and MER Recordings**

| **Dystonia Gene** | **Number of Patients** | **Number of MERs** | **Number of Stable Neurons** |
| --- | --- | --- | --- |
| *AOPEP* | 1 | 69 | 45 |
| *KMT2B* | 2 | 102 | 94 |
| *GNAL* | 2 | 93 | 50 |
| *PANK2* | 3 | 231 | 119 |
| *PLA2G6* | 1 | 284 | 169 |
| *SGCE* | 4 | 620 | 364 |
| *THAP1* | 4 | 176 | 74 |
| *TOR1A* | 9 | 1121 | 434 |
| *VPS16* | 5 | 497 | 345 |
|  | **31** | **3193** | **1694** |

**Supplementary Figure 1. The DBS Lead placements of our dystonia patient cohort.** The optimal trajectory for stimulation, based on multiple MER trajectories, was selected for each hemisphere, and the lead electrode was placed along this trajectory. The reconstruction of GPi, GPe, and recording depths are defined using the Distal Atlas in Montreal Neurological Institute space (p > 0.5 thresholds for definitions of nuclei borders) based on electrode reconstructions made with Lead-DBS and using postoperative brain MRI or CT images

**
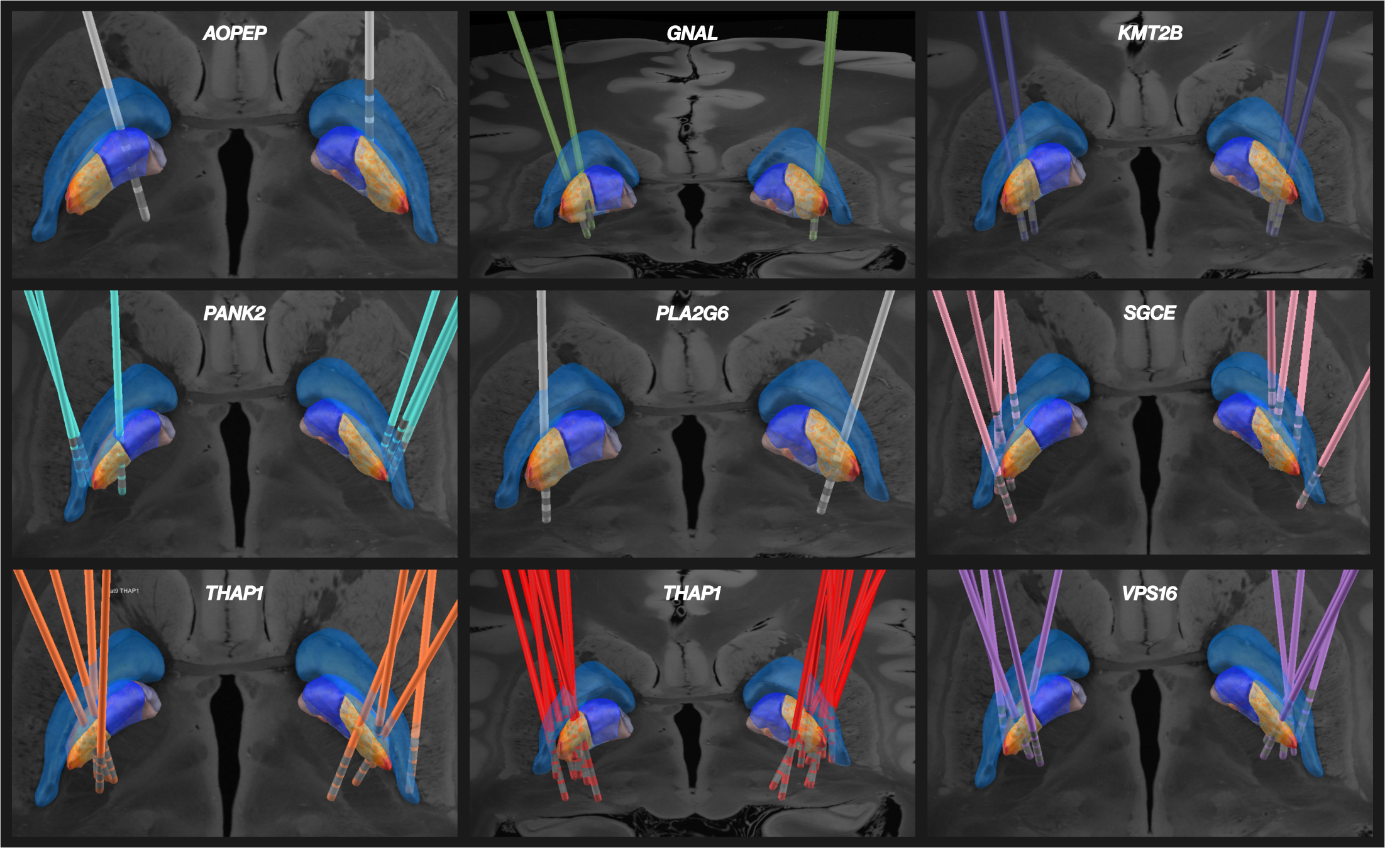
**

**Supplementary Figure 2. Example of microelectrode recordings across the depths of the central trajectory.** Single-unit activities were highlighted on the raw recordings and color-coded based on their firing pattern (tonic, irregular, or bursty).

**
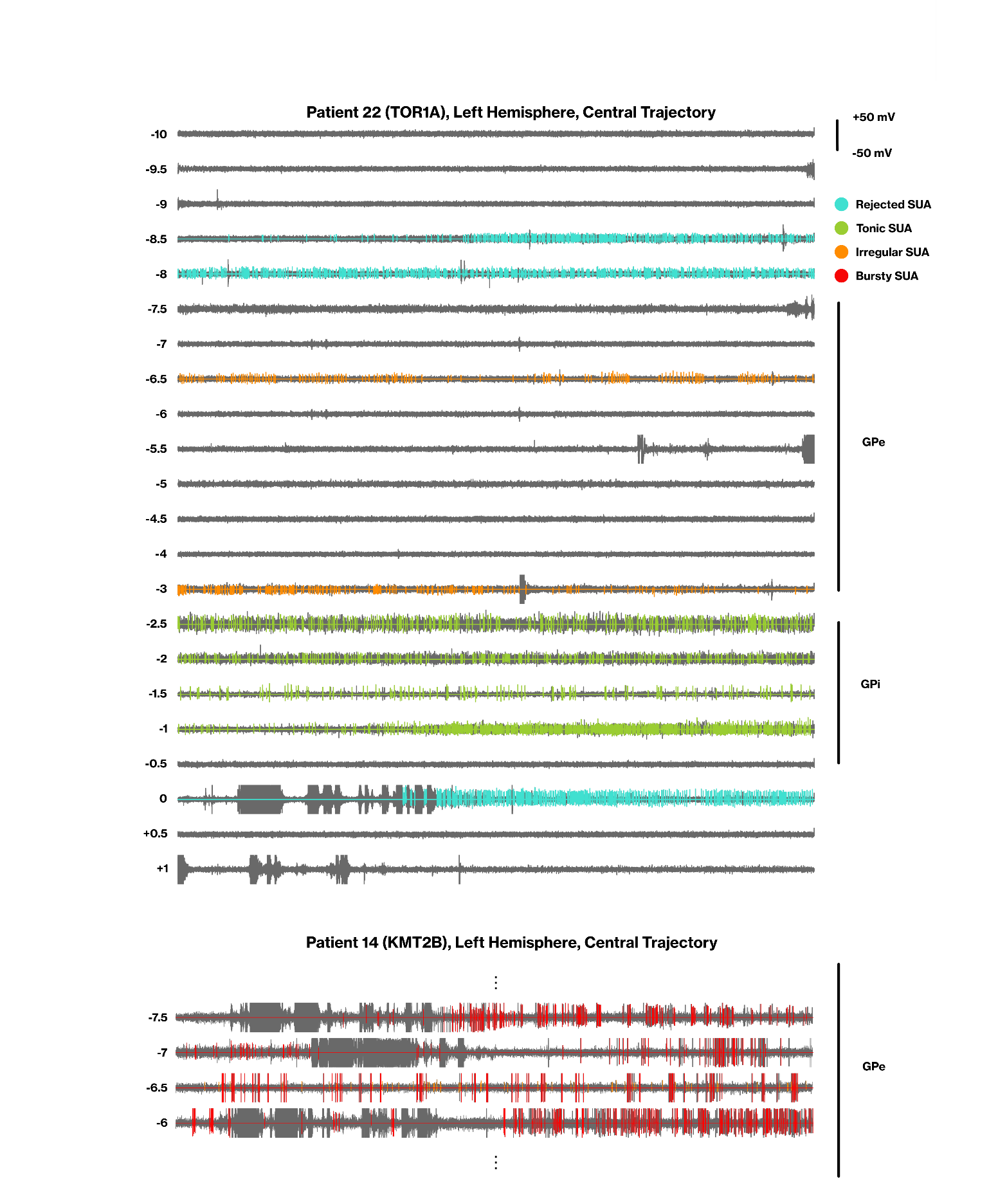
**

**Supplementary Figure 3. Example of tonic, irregular, and bursting neurons with their corresponding raw recordings, action potentials, instantaneous firing rates, raster plots, and interspike interval (ISI) histograms.**

**
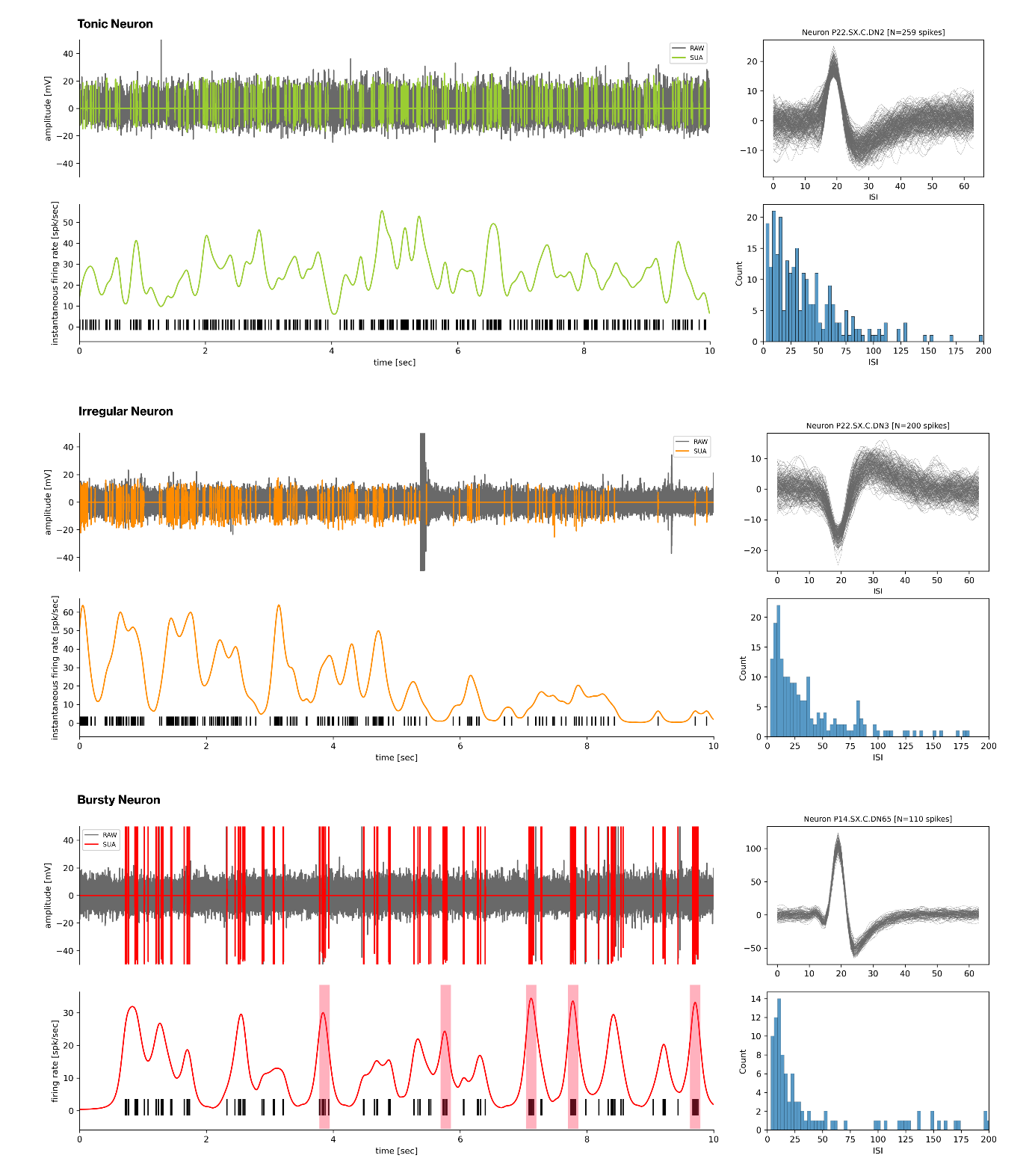
**

# Neural Feature Generation

We conducted extensive neural feature extraction from MERs to characterize pallidal neural activity across different genetic variants. The extracted features included firing rate, firing irregularity, burstiness, neural pauses, and oscillatory behaviors. These neural characteristics provide crucial information about the neural dynamics within the globus pallidum and their potential associations with specific genetic forms of dystonia.

## Firing rate and firing regularity

Despite its usefulness, the mean firing rate does not provide a complete picture of the temporal firing characteristics of a neuron. Consequently, researchers generally rely on other types of schemes for a better understanding of neural functions. An example of these schemes is the instantaneous firing rate of a neuron. We adopted the adaptive kernel smoother^107^ (BAKS) test to estimate the instantaneous firing rate. BAKS uses a kernel smoothing technique with adaptive bandwidth. The neural process dynamics are encapsulated by the adaptive bandwidth for the selected kernel function. In this case, if the firing rate increases in the spike train, the bandwidth of the kernel function will be decreased by the BAKS algorithm. The adaptation of the bandwidth paves the way for defining accurate estimations of the instantaneous firing rate under slow and fast-spiking dynamics. The kernel bandwidth is recognized by BAKS as a random variable with a prior distribution. It uses an empirical Bayesian method to change the posterior bandwidth. We set α = 4 and β = N^(4/5)^ as two parameters of BAKS and estimated the instantaneous firing rate for each SUA.

**Supplementary Table 4. Firing rate- and firing regularity-related neural features, their descriptions, and formulations.**

| **Neural Feature** | **Definition** | **Formulation** |
| --- | --- | --- |
| firing rate | It is the simplest and most widely used neural feature for neural data analysis. It is directly connected to the outcome of neural functioning. | the rate parameter (λ) of the fitted gamma distribution for the ISI distribution |
| firing regularity | This metric quantifies the regularity of the spiking of a neuron and provides intuition about the general firing characteristic of the neuron. Firing regularity^108^ metrics can help us classify the neurons into subcategories based on their firing characteristic. | $firing regularity=log(\kappa)$  $firing pattern=\left\{ \begin{aligned} \begin{aligned} \log\left( \kappa\right)\leq-0.3, bursting \\ \log\left( \kappa\right)\geq0.3, tonic \end{aligned} \\ -0.3>log \left( \kappa\right)>0.3, additional criterion \end{aligned} \right\}$  $additional criterion=\left\{ \begin{aligned} 70\% samples>\vert\lambda\pm0.5\lambda\vert, bursting \\ 70\% samples<\vert\lambda\pm0.5\lambda\vert, irregular \end{aligned} \right\}$ |
| cv | The coefficient of variation in spike trains is evaluated to quantify the width of the ISI distribution.^109^ It acts as a sort of measure for spike train irregularity. | $cv=\frac{\sqrt[2]{variance(ISI)}}{mean(ISI)}$ |
| lv | It is a metric originally defined to determine the intrinsic temporal dynamics of neuron spike trains. Lv compares temporal variations with their local rates, and it is specifically defined for nonstationary processes. In the equation, each $\tau$ value indicates the timing of an observed spike where N parameters represent the total number of spikes in a spike train.^110^ | $lv= \frac{3}{N-2}\sum_{n=0}^{N-1} \left( \frac{\left( \tau_{n+1}-\tau_{n} \right)-(\tau_{n}-\tau_{n-1})}{\left( \tau_{n+1}-\tau_{n} \right)-(\tau_{n}-\tau_{n-1})} \right)^{2}$ |
| asymmetry index | A neural metric to measure spiking regularity which is based on ISI distribution statistics. | $asymmetry index=\frac{mode(ISI)}{mean(ISI)}$ |
| isi mean | The mean value of ISI represents the average temporal distance between two subsequent spikes of a neural structure. It is closely correlated with the mean firing rate. | $ISI_{mean}= shape_{param}\times scale_{param}$ |
| isi std | The standard deviation was calculated to define the dispersion around the mean of the ISI distribution. It indicates the uncertainty of the duration between two subsequent spikes in spike trains. | $ISI_{std}= \sqrt{shape_{param} \times{scale_{param}}^{2}}$ |
| isi skewness | Skewness represents how ISI values are symmetrically distributed around the distribution mean. | $ISI_{skewness}= \frac{2}{\sqrt{shape_{param}}}$ |
| isi correlation coefficient | Correlations between interspike intervals occur due to a combination of intrinsic mechanisms and the temporal properties of the input stimulus. ISI correlation can provide indirect information from bursting to periodical phase-locked firing.^111^ ISI correlation is computed with the serial correlation coefficient formula. | ${ISI}_{rho}= SCC =$  $\frac{\frac{1}{N}\sum_{i=1}^{N} \left( ISI_{i}-\bar{ISI_{i}} \right)\times(ISI_{i+k}-\bar{ISI_{i+k}})}{\frac{1}{N}\sum_{i=1}^{N} {(ISI_{i}-\bar{ISI_{i}} )}^{2}}$ |

## Neural Bursting

Bursting is a dynamic state in which a neuron repeatedly fires discrete groups or bursts of spikes in a small temporal region. There are a vast number of burst detections available in the literature. We used an approach called Rank Surprise^112^ for the detection of bursting activity. In this method, the ISI values are calculated and ordered with ascending trends. The ordered ISI values are given a rank. If the rank surprise value is higher than the user-defined threshold, the temporal region will be registered as a busting activity. These regions are subtracted from the spike train, and the new iteration for finding the bursting region is initiated until there is no bursting region detected by the algorithm. Two parameters are user-defined: the largest ISI allowed in a burst (limit) and the minimum RS (threshold) that a burst must be considered valid. We selected 75% as the largest ISI allowed in a burst and 0.01 as the minimum significance level for the selected burst region.

**Supplementary Table 5. Neural burst-related features, their descriptions, and formulations.**

| burst spike proportion | It represents the proportion of spikes that are located inside the burst intervals compared to all existing spikes in the spike train. | $\boldsymbol{burst spike proportion=}$  $\frac{\sum_{\boldsymbol{n=1}}^{\boldsymbol{N}_{\boldsymbol{bursts}}} \boldsymbol{coun}\boldsymbol{t}_{\boldsymbol{n}}\boldsymbol{(spikes)}}{\boldsymbol{count(all spikes)}}$ |
| --- | --- | --- |
| burst average spikes | The feature indicates the average number of spikes within the bursting interval. | $burst average spikes=$  $\frac{1}{N_{bursts}}\sum_{n=1}^{N_{bursts}} count_{n}(spikes)$ |
| burst index | The burst index^12^ is a metric that is defined for the assessment of possible changes in firing patterns. It is a widely used feature for cortical neural analysis. | $burst index= \frac{mean(ISI)}{mod(ISI)}$ |
| inter burst duration | It indicates the average temporal distance between subsequent bursts. | $inter burst duration=$  $\frac{1}{N_{bursts}-1}\sum_{n=1}^{N_{bursts}} {(\tau}_{burst\_start_{n+1}}-\tau_{burst\_finish_{n}})$ |
| burst frequency | The mean firing rate during the bursting intervals. | $burst frequency=$  $\frac{1}{N_{bursts}}\sum_{n=1}^{N_{bursts}} \frac{count_{n}(spikes)}{{(\tau}_{finish_{n}}-\tau_{start_{n}})}$ |
| burst duration | The mean duration of bursting activity within the spike train. | $burst duration=$  $\frac{1}{N_{bursts}}\sum_{n=1}^{N_{bursts}} {(\tau}_{burst\_finish_{n}}-\tau_{burst\_start_{n}})$ |
| burst count | The number of spikes shows burstiness. | $burst count= \sum_{n=1}^{N_{bursts}} spike$ |

## Neural Oscillations

Neural oscillations are rhythmic patterns of neural activity in the CNS. The generation of neural oscillations can be mediated by multiple factors, such as intrinsic properties of the oscillating neurons or network-wide interactions between other neurons. From this perspective, the rhythmic activity of neurons plays a major role in neural processing and behavior.^113^ In addition to their role in the normal functioning of neurons, neural oscillations can also occur under pathological conditions. Pathological periodic bursting activity within the corticothalamic-basal ganglia network has been identified as a key hallmark of Parkinson's disease.^114^

To detect neural oscillations, we utilized the widely used oscillation score^115^ approach. This technique involves calculating autocorrelation histograms on spike trains to estimate the strength of neuronal oscillations at the single-neuron level. To establish a confidence measure for the oscillation scores in our single-trial experiments, we employed a bootstrap test with 100 iterations. In each iteration, we randomly shuffled the spike train while maintaining the number of spikes and the refractory period. Subsequently, we computed the oscillation score for the shuffled spike train. By repeating this process for 100 iterations, we obtained a null distribution of oscillation scores, representing random activity for the selected SUA. To determine the significance level for the computed oscillation score within a specific frequency band, we defined the boundaries of the 95th percentile of the null distribution (2.5% for the lower boundary and 97.5% for the upper boundary). If the original oscillation score exceeded the threshold defined by the upper boundary, we considered the observed oscillatory activity to be significant. Bootstrapping allowed us to assess the significance of the detected neural oscillations and provided a reliable measure for our analyses. Finally, we saved the oscillation frequencies for each frequency band.

## Neural Pauses

In contrast to neural bursts, neural pauses represent periods of hypoactivity in neurons, where spiking activity disappears. We consider neural pauses to be an essential mechanism for observing the behavior of SUA. These pauses can provide valuable insights into the dynamics of neural firing. Indeed, neural pauses have been previously utilized to distinguish neural activity within the external segment of the globus pallidum (GPe) between primary and secondary types of dystonia.^116^ For the detection of pauses, we used the Poisson Surprise^117^ method. After detecting the temporal location of pauses in spike trains, we measured several metrics, such as pause frequency, pause count, pause duration, etc.

**Supplementary Table 6. Neural pauses-related features, their descriptions, and formulations.**

| pause index | number of ISI higher than 50ms divided by the number of ISI lower than 50ms.^118^ | $\boldsymbol{pause index =}\frac{\sum_{\boldsymbol{i=1}}^{\boldsymbol{N}} \boldsymbol{\delta}_{\boldsymbol{ISI}_{\boldsymbol{i}}\boldsymbol{\geq50}}}{\sum_{\boldsymbol{i=1}}^{\boldsymbol{N}} \boldsymbol{\delta}_{\boldsymbol{ISI}_{\boldsymbol{i}}\boldsymbol{<50}}}$ |
| --- | --- | --- |
| pause ratio | cumulative of ISI higher than 50ms divided by the number of ISI lower than 50ms. | $pause ratio = \frac{\sum_{i=1}^{N} {ISI}_{i}\geq50}{\sum_{i=1}^{N} \delta_{{ISI}_{i}<50}}$ |
| pause frequency | The mean firing rate during the pause intervals. | $pause frequency=$  $\frac{1}{N_{pauses}}\sum_{n=1}^{N_{pauses}} \frac{count_{n}(spikes)}{{(\tau}_{finish_{n}}-\tau_{start_{n}})}$ |
| pause duration | The mean duration of pauses within the spike train. | $pause_{duration}=$  $\frac{1}{N_{pauses}}\sum_{n=1}^{N_{pauses}} {(\tau}_{pause\_finish_{n}}-\tau_{pause\_start_{n}})$ |
| pause count | The number of spikes in the pause regions. | $pause count= \sum_{n=1}^{N_{pause\_spike}} 1$ |
| pause time proportion | Amount of time spent in the pause state compared to the total recording duration | $pause time proportion=$  $\frac{\sum_{n=1}^{N_{pauses}} {(\tau}_{finish_{n}}-\tau_{start_{n}})}{\tau_{recording}}$ |
| pause spike proportion | The ratio between spikes that occurred in the pause state and the total number of spikes | $pause spike proportion= \frac{N_{pauses}}{N_{spikes}}$ |

# Effect Size Interpretation

For the Kruskal‒Wallis and Mann‒Whitney U tests, we measured Cohen’s d effect size and followed general guidelines for interpreting effect size^119,120^:

- 0.2 ≤small effect<0.5
- 0.5≤medium effect<0.8
- 0.8≥large effect

For the chi-square test, we utilized Cramer’s v as the effect size metric, which falls within the range of 0 to 1. We followed these ranges to interpret the effect size^120^:

- small effect<0.15
- 0.15≤medium effect<0.25
- large effect ≥0.25

# Neural Feature Categories

We categorized the neural features based on their association with a specific neural phenomenon for several analyses.

**Supplementary Table 7. Neural features and the neural phenomena associated with them.**

| **Neural Feature** | **Regularity** | **Bursts** | **Pauses** | **Oscillations** | **Pattern** |
| --- | --- | --- | --- | --- | --- |
| firing rate |  |  |  |  |  |
| firing regularity | X |  |  |  |  |
| cv | X |  |  |  |  |
| lv | X |  |  |  |  |
| isi mean |  |  |  |  |  |
| isi std | X |  |  |  |  |
| isi skewness | X |  |  |  |  |
| isi rho | X |  |  |  |  |
| pause index |  |  | X |  |  |
| pause ratio |  |  | X |  |  |
| asymmetry index | X |  |  |  |  |
| tonic | X |  |  |  |  |
| irregular | X |  |  |  | X |
| burst index |  | X |  |  |  |
| burst average spikes |  | X |  |  |  |
| burst spike proportion |  | X |  |  |  |
| burst duration |  | X |  |  |  |
| inter burst duration |  | X |  |  |  |
| burst frequency |  | X |  |  |  |
| burst count |  | X |  |  |  |
| bursting |  | X |  |  | X |
| pause frequency |  |  | X |  |  |
| pause duration |  |  | X |  |  |
| pause time proportion |  |  | X |  |  |
| pause spike proportion |  |  | X |  |  |
| pause count |  |  | X |  |  |
| delta oscillation frequency |  |  |  | X |  |
| theta oscillation frequency |  |  |  | X |  |
| alpha oscillation frequency |  |  |  | X |  |
| beta oscillation frequency |  |  |  | X |  |
| gamma oscillation frequency |  |  |  | X |  |
| delta band oscillatory |  |  |  | X |  |
| theta band oscillatory |  |  |  | X |  |
| alpha band oscillatory |  |  |  | X |  |
| beta band oscillatory |  |  |  | X |  |
| gamma band oscillatory |  |  |  | X |  |
| oscillatory |  |  |  | X | X |

**Supplementary Figure 4 – Details of the two stratification strategies developed for dystonia genes.** (A) Pooling of neurons from patients with pathogenic mutations in the same dystonia gene. (B) Schematic representation of stratification by thresholding approach. (C) Schematic representation of stratification by distance approach, which categorizes dystonia genes based on the cumulative distance between neural feature value distributions.


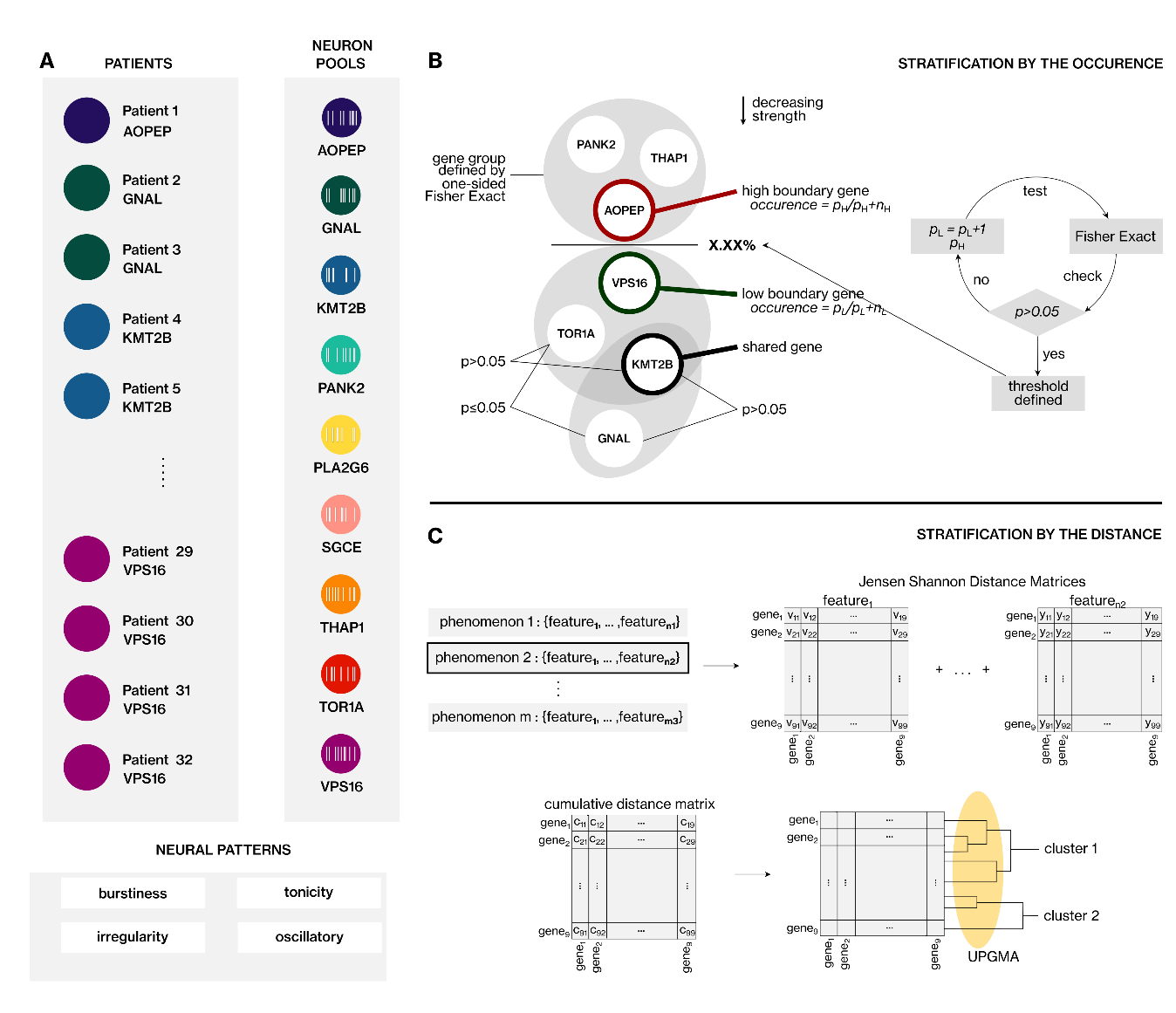


# Computational pipeline for neural decoders

We selected the random forest tree and gradient boosting approaches, along with a voting classifier that combines the predictions of these two techniques through a soft voting mechanism. We applied a stratified 5-fold cross-validation approach in which, in each fold, we trained the models with 80% of the neurons, and then, we used the remaining 20% of the neurons to test the performances of the trained models. To assess and contrast the performance of the trained models, we relied on metrics such as the balanced accuracy score, weighted area under the curve (AUC), and weighted F1 score. We averaged the scores of these three metrics fivefold to define the final performance of each decoder. Furthermore, we conducted a comprehensive validation of our trained models using a bootstrap test with 100 iterations. In each iteration, we introduced randomness by shuffling the genetic variant information (the label attribute); subsequently, we determined the average performance of the models as described in the previous paragraph. By repeating this procedure across all iterations, we generated a distribution of values (consisting of 100 scores) representing the model's performance under random circumstances. These distributions offer insights into the models' performances in the context of random events. Subsequently, we calculated the 95th percentile of this random-event distribution for each metric, establishing an upper threshold for the models' classification performances in random events. We expected that the trained models/decoders would surpass these defined thresholds when applied to the actual dataset. In instances where this did not occur, it would indicate that the model had learned the random fluctuations or errors in the neural data rather than the distinct patterns associated with each genetic variant.

## One-vs-one (OVO) Decoding

Given the intricate nature of decoding neural activity at the SUA level, our initial focus was on classifying the neural patterns between dystonia-causing gene pairs. In total, we identified 36 possible gene pairs. For OVO decoding, we selected neural features that exhibited at least a medium effect size (*Cohen's d* ≥ 0.8 or *Cramer's v* ≥ 0.25), which signifies their practical utility, for the variants in each pair. We included all features in the model training for gene pairs where this condition was not met. We presented the performance of the random forest (RF) tree classifier, gradient boost classifier, and voting classifier in terms of balanced accuracy, weighted area under the curve (AUC), and weighted F1 (Supplementary Table 8).

**Supplementary Table 8. The one-vs-one (OVO) decoding performances**

| **Gene Pair** | | **Random Forest Classifier** | | | **Gradient Boost Classifier** | | | **Voting Classifier** | | |
| --- | --- | --- | --- | --- | --- | --- | --- | --- | --- | --- |
|  |  | **balanced**  **accuracy** | **weighted**  **AUC** | **weighted**  **F1** | **balanced**  **accuracy** | **weighted**  **AUC** | **weighted**  **F1** | **balanced**  **accuracy** | **weighted**  **AUC** | **weighted**  **F1** |
| *AOPEP* | *GNAL* | 0.78 | 0.84 | 0.78 | 0.74 | 0.80 | 0.74 | 0.75 | 0.82 | 0.75 |
| *AOPEP* | *KMT2B* | 0.83 | 0.85 | 0.82 | 0.81 | 0.84 | 0.83 | 0.81 | 0.86 | 0.82 |
| *AOPEP* | *PANK2* | 0.70 | 0.73 | 0.74 | 0.66 | 0.73 | 0.71 | 0.66 | 0.74 | 0.71 |
| *AOPEP* | *PLA2G6* | 0.81 | 0.86 | 0.85 | 0.81 | 0.90 | 0.87 | 0.80 | 0.87 | 0.87 |
| *AOPEP* | *SGCE* | 0.82 | 0.89 | 0.90 | 0.83 | 0.93 | 0.93 | 0.85 | 0.90 | 0.94 |
| *AOPEP* | *THAP1* | 0.66 | 0.77 | 0.67 | 0.66 | 0.71 | 0.67 | 0.65 | 0.73 | 0.66 |
| *AOPEP* | *TOR1A* | 0.81 | 0.89 | 0.89 | 0.78 | 0.89 | 0.91 | 0.78 | 0.89 | 0.92 |
| *AOPEP* | *VPS16* | 0.75 | 0.81 | 0.85 | 0.72 | 0.82 | 0.87 | 0.73 | 0.81 | 0.87 |
| *GNAL* | *KMT2B* | 0.61 | 0.64 | 0.65 | 0.58 | 0.63 | 0.63 | 0.61 | 0.64 | 0.66 |
| *GNAL* | *PANK2* | 0.76 | 0.82 | 0.78 | 0.73 | 0.76 | 0.76 | 0.75 | 0.80 | 0.77 |
| *GNAL* | *PLA2G6* | 0.51 | 0.53 | 0.57 | 0.50 | 0.55 | 0.58 | 0.51 | 0.53 | 0.60 |
| *GNAL* | *SGCE* | 0.61 | 0.62 | 0.73 | 0.59 | 0.61 | 0.73 | 0.61 | 0.61 | 0.73 |
| *GNAL* | *THAP1* | 0.78 | 0.84 | 0.79 | 0.68 | 0.76 | 0.69 | 0.70 | 0.81 | 0.71 |
| *GNAL* | *TOR1A* | 0.60 | 0.68 | 0.76 | 0.56 | 0.65 | 0.80 | 0.61 | 0.67 | 0.81 |
| *GNAL* | *VPS16* | 0.63 | 0.68 | 0.73 | 0.60 | 0.67 | 0.73 | 0.62 | 0.68 | 0.72 |
| *KMT2B* | *PANK2* | 0.72 | 0.79 | 0.72 | 0.72 | 0.79 | 0.72 | 0.71 | 0.78 | 0.72 |
| *KMT2B* | *PLA2G6* | 0.61 | 0.63 | 0.59 | 0.58 | 0.63 | 0.54 | 0.60 | 0.63 | 0.58 |
| *KMT2B* | *SGCE* | 0.55 | 0.55 | 0.74 | 0.55 | 0.55 | 0.74 | 0.55 | 0.55 | 0.74 |
| *KMT2B* | *THAP1* | 0.79 | 0.88 | 0.79 | 0.81 | 0.86 | 0.81 | 0.81 | 0.88 | 0.81 |
| *KMT2B* | *TOR1A* | 0.59 | 0.62 | 0.69 | 0.52 | 0.54 | 0.68 | 0.55 | 0.57 | 0.69 |
| *KMT2B* | *VPS16* | 0.60 | 0.64 | 0.70 | 0.59 | 0.63 | 0.68 | 0.59 | 0.64 | 0.69 |
| *PANK2* | *PLA2G6* | 0.70 | 0.78 | 0.70 | 0.70 | 0.79 | 0.70 | 0.71 | 0.79 | 0.71 |
| *PANK2* | *SGCE* | 0.76 | 0.82 | 0.78 | 0.76 | 0.84 | 0.78 | 0.76 | 0.84 | 0.79 |
| *PANK2* | *THAP1* | 0.62 | 0.65 | 0.59 | 0.50 | 0.56 | 0.52 | 0.57 | 0.59 | 0.58 |
| *PANK2* | *TOR1A* | 0.66 | 0.73 | 0.73 | 0.63 | 0.71 | 0.75 | 0.63 | 0.72 | 0.75 |
| *PANK2* | *VPS16* | 0.61 | 0.68 | 0.67 | 0.57 | 0.62 | 0.67 | 0.58 | 0.67 | 0.68 |
| *PLA2G6* | *SGCE* | 0.56 | 0.61 | 0.61 | 0.55 | 0.55 | 0.59 | 0.56 | 0.57 | 0.60 |
| *PLA2G6* | *THAP1* | 0.74 | 0.80 | 0.77 | 0.68 | 0.78 | 0.74 | 0.69 | 0.80 | 0.74 |
| *PLA2G6* | *TOR1A* | 0.65 | 0.69 | 0.72 | 0.61 | 0.63 | 0.69 | 0.63 | 0.66 | 0.71 |
| *PLA2G6* | *VPS16* | 0.62 | 0.66 | 0.65 | 0.60 | 0.62 | 0.64 | 0.60 | 0.63 | 0.65 |
| *SGCE* | *THAP1* | 0.78 | 0.85 | 0.83 | 0.72 | 0.84 | 0.83 | 0.71 | 0.85 | 0.82 |
| *SGCE* | *TOR1A* | 0.65 | 0.71 | 0.65 | 0.62 | 0.68 | 0.62 | 0.62 | 0.70 | 0.62 |
| *SGCE* | *VPS16* | 0.63 | 0.70 | 0.63 | 0.64 | 0.69 | 0.64 | 0.64 | 0.70 | 0.64 |
| *THAP1* | *TOR1A* | 0.75 | 0.81 | 0.81 | 0.65 | 0.77 | 0.82 | 0.68 | 0.79 | 0.82 |
| *THAP1* | *VPS16* | 0.67 | 0.74 | 0.75 | 0.60 | 0.71 | 0.76 | 0.59 | 0.73 | 0.75 |
| *TOR1A* | *VPS16* | 0.61 | 0.64 | 0.61 | 0.59 | 0.63 | 0.60 | 0.59 | 0.65 | 0.60 |

## One-vs-rest (OVR) decoding

Furthermore, we also tried to successfully decode neurons associated with each genetic dystonia syndrome in contrast to the others. Employing a neural feature selection scheme similar to OVO decoding, we initiated Mann‒Whitney U tests with Holm–Bonferroni correction in an OVR fashion. These tests aimed to pinpoint neural features distinguishing the neurons of the selected genetic dystonia syndromes from the rest, with corresponding p values provided in Supplementary Table 7. For OVR decoding, we exclusively employed the random forest classifier method, as different algorithms yielded comparable results in OVO decoding. The outcomes are presented in terms of balanced accuracy, weighted area under the curve (AUC), weighted F1, and recall scores, with a particular emphasis on recall, given the focus on targeting neurons of the specific genetic syndrome rather than the remaining syndromes (Supplementary Table 8).

**Supplementary Figure 5. One-vs-rest statistical comparisons of pallidal neurons in patients with genetic dystonia syndromes**

**
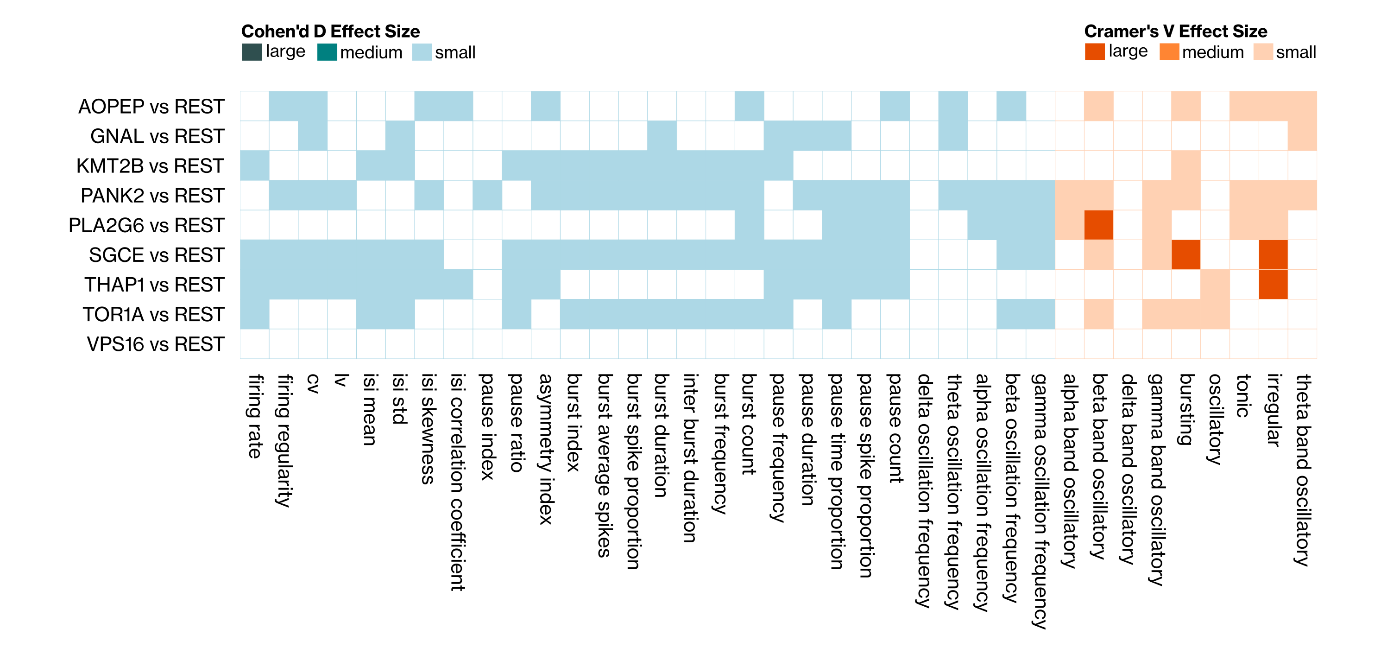
**

**Supplementary Table 9. The one-vs-rest (OVR) decoding performances**

| **Neural Decoder** | **balanced**  **accuracy** | **weighted**  **AUC** | **weighted**  **F1** |
| --- | --- | --- | --- |
| *AOPEP* vs REST | 0.756284 | 0.820838 | 0.85273 |
| *KMT2B* vs REST | 0.540599192 | 0.648056565 | 0.744992797 |
| *GNAL* vs REST | 0.557050439 | 0.562206689 | 0.813473037 |
| *PANK2* vs REST | 0.686158 | 0.740158 | 0.721691 |
| *PLA2G6* vs REST | 0.607557056 | 0.629575991 | 0.810978847 |
| *SGCE* vs REST | 0.6082774 | 0.666171851 | 0.695081905 |
| *THAP1* vs REST | 0.716332 | 0.77672 | 0.789802 |
| *TOR1A* vs REST | 0.584547 | 0.614407 | 0.560128 |
| *VPS16* vs REST | 0.56385 | 0.641069 | 0.734393 |

**REFERENCES**

1. Thomsen M, Lange LM, Klein C, Lohmann K. MDSGene : Extending the List of Isolated Dystonia Genes by *VPS16* , *EIF2AK2* , and *AOPEP* . *Mov Disord*. 2023;38(3):507-508. doi:10.1002/mds.29327

2. Garavaglia B, Vallian S, Romito LM, et al. AOPEP variants as a novel cause of recessive dystonia: Generalized dystonia and dystonia-parkinsonism. *Parkinsonism Relat Disord*. 2022;97:52-56. doi:10.1016/j.parkreldis.2022.03.007

3. Zech M, Kumar KR, Reining S, et al. Biallelic *AOPEP* Loss‐of‐Function Variants Cause Progressive Dystonia with Prominent Limb Involvement. *Mov Disord*. 2022;37(1):137-147. doi:10.1002/mds.28804

4. Fevga C, Ferraro F, Breedveld GJ, Savant Sankhla C, Bonifati V. *AOPEP* Homozygous Loss‐of‐Function Variant in an Indian Patient with Early‐Onset Generalized Dystonia. *Mov Disord*. 2022;37(4):874-875. doi:10.1002/mds.28928

5. Menden B, Gutschalk A, Wunderlich G, Haack TB. Expanded Genetic Spectrum and Variable Disease Onset in *AOPEP* ‐Associated Dystonia. *Mov Disord*. 2022;37(5):1113-1115. doi:10.1002/mds.29021

6. Lin J, Li C, Cui Y, et al. Mutation screening of AOPEP variants in a large dystonia cohort. *J Neurol*. 2023;270(6):3225-3233. doi:10.1007/s00415-023-11665-7

7. Fuchs T, Saunders-Pullman R, Masuho I, et al. Mutations in GNAL cause primary torsion dystonia. *Nat Genet*. 2013;45(1):88-92. doi:10.1038/ng.2496

8. Tisch S, Kumar KR. Pallidal Deep Brain Stimulation for Monogenic Dystonia: The Effect of Gene on Outcome. *Front Neurol*. 2021;11:630391. doi:10.3389/fneur.2020.630391

9. Dobričić V, Kresojević N, Westenberger A, et al. De novo mutation in the *GNAL* gene causing seemingly sporadic dystonia in a Serbian patient: DE NOVO *GNAL* (DYT25) MUTATION. *Mov Disord*. 2014;29(9):1190-1193. doi:10.1002/mds.25876

10. Zech M, Boesch S, Sycha T, Mueller J, Poewe W, Winkelmann J. *TOR1A, THAP1* , and *GNAL* mutational screening in Austrian patients with primary isolated dystonia: Letter to the Editors. *Mov Disord*. 2015;30(13):1853-1854. doi:10.1002/mds.26458

11. Miao J, Wan XH, Sun Y, Feng JC, Cheng FB. Mutation screening of GNAL gene in patients with primary dystonia from Northeast China. *Parkinsonism Relat Disord*. 2013;19(10):910-912. doi:10.1016/j.parkreldis.2013.05.011

12. Vemula SR, Puschmann A, Xiao J, et al. Role of Gα(olf) in familial and sporadic adult-onset primary dystonia. *Hum Mol Genet*. 2013;22(12):2510-2519. doi:10.1093/hmg/ddt102

13. Saunders-Pullman R, Fuchs T, San Luciano M, et al. Heterogeneity in primary dystonia: Lessons from *THAP1* , *GNAL* , and *TOR1A* in Amish-Mennonites: *THAP1* , *GNAL* , AND *TOR1A* in Amish-Mennonites. *Mov Disord*. 2014;29(6):812-818. doi:10.1002/mds.25818

14. Ziegan J, Wittstock M, Westenberger A, et al. Novel *GNAL* mutations in two German patients with sporadic dystonia: LETTERS: NEW OBSERVATIONS. *Mov Disord*. 2014;29(14):1833-1834. doi:10.1002/mds.26066

15. Carecchio M, Panteghini C, Reale C, et al. Novel GNAL mutation with intra-familial clinical heterogeneity: Expanding the phenotype. *Parkinsonism Relat Disord*. 2016;23:66-71. doi:10.1016/j.parkreldis.2015.12.012

16. LeDoux MS, Vemula SR, Xiao J, et al. Clinical and genetic features of cervical dystonia in a large multicenter cohort. *Neurol Genet*. 2016;2(3):e69. doi:10.1212/NXG.0000000000000069

17. Sarva H, Trosch R, Kiss ZHT, et al. Deep Brain Stimulation in Isolated Dystonia With a *GNAL* Mutation. *Mov Disord*. 2019;34(2):301-303. doi:10.1002/mds.27585

18. Domingo A, Yadav R, Ozelius LJ. Isolated dystonia: clinical and genetic updates. *J Neural Transm*. 2021;128(4):405-416. doi:10.1007/s00702-020-02268-x

19. Fan S, Cao Q, Peng B, et al. A new mutation in the GNAL gene in familial dystonia presenting with mental symptoms. *Neurol Sci*. 2022;43(7):4547-4549. doi:10.1007/s10072-022-06060-5

20. Zech M, Boesch S, Maier EM, et al. Haploinsufficiency of KMT2B, Encoding the Lysine-Specific Histone Methyltransferase 2B, Results in Early-Onset Generalized Dystonia. *Am J Hum Genet*. 2016;99(6):1377-1387. doi:10.1016/j.ajhg.2016.10.010

21. UK10K Consortium, Deciphering Developmental Disorders Study, NIHR BioResource Rare Diseases Consortium, et al. Mutations in the histone methyltransferase gene KMT2B cause complex early-onset dystonia. *Nat Genet*. 2017;49(2):223-237. doi:10.1038/ng.3740

22. Carecchio M, Invernizzi F, Gonzàlez‐Latapi P, et al. Frequency and phenotypic spectrum of *KMT2B* dystonia in childhood: A single‐center cohort study. *Mov Disord*. 2019;34(10):1516-1527. doi:10.1002/mds.27771

23. Li X yao, Dai L fang, Wan X hua, et al. Clinical phenotypes, genotypes and treatment in Chinese dystonia patients with KMT2B variants. *Parkinsonism Relat Disord*. 2020;77:76-82. doi:10.1016/j.parkreldis.2020.06.002

24. Cif L, Demailly D, Lin JP, et al. *KMT2B* -related disorders: expansion of the phenotypic spectrum and long-term efficacy of deep brain stimulation. *Brain*. 2020;143(11):3242-3261. doi:10.1093/brain/awaa304

25. Zech M, Lam DD, Winkelmann J. Update on KMT2B-Related Dystonia. *Curr Neurol Neurosci Rep*. 2019;19(11):92. doi:10.1007/s11910-019-1007-y

26. Zech M, Jech R, Havránková P, et al. *KMT2B* rare missense variants in generalized dystonia: *KMT2B* Missense Variants in Dystonia. *Mov Disord*. 2017;32(7):1087-1091. doi:10.1002/mds.27026

27. Dafsari HS, Sprute R, Wunderlich G, et al. Novel mutations in KMT2B offer pathophysiological insights into childhood-onset progressive dystonia. *J Hum Genet*. 2019;64(8):803-813. doi:10.1038/s10038-019-0625-1

28. Aksoy A, Yayıcı Köken Ö, Ceylan AC, Toptaş Dedeoğlu Ö. ***KMT2B***-Related Dystonia: Challenges in Diagnosis and Treatment. *Mol Syndromol*. 2022;13(2):159-164. doi:10.1159/000518974

29. Kumar KR, Davis RL, Tchan MC, et al. Whole genome sequencing for the genetic diagnosis of heterogenous dystonia phenotypes. *Parkinsonism Relat Disord*. 2019;69:111-118. doi:10.1016/j.parkreldis.2019.11.004

30. Cao Z, Yao H, Bao X, et al. DYT28 Responsive to Pallidal Deep Brain Stimulation. *Mov Disord Clin Pract*. 2020;7(1):97-99. doi:10.1002/mdc3.12862

31. Kawarai T, Miyamoto R, Nakagawa E, et al. Phenotype variability and allelic heterogeneity in KMT2B-Associated disease. *Parkinsonism Relat Disord*. 2018;52:55-61. doi:10.1016/j.parkreldis.2018.03.022

32. Thomas M, Hayflick SJ, Jankovic J. Clinical heterogeneity of neurodegeneration with brain iron accumulation (Hallervorden-Spatz syndrome) and pantothenate kinase-associated neurodegeneration: Hallervorden-Spatz Syndrome and PKAN. *Mov Disord*. 2004;19(1):36-42. doi:10.1002/mds.10650

33. Umemura A, Jaggi JL, Dolinskas CA, Stern MB, Baltuch GH. Pallidal deep brain stimulation for longstanding severe generalized dystonia in Hallervorden—Spatz syndrome: Case report. *J Neurosurg*. 2004;100(4):706-709. doi:10.3171/jns.2004.100.4.0706

34. Hogarth P. Neurodegeneration with Brain Iron Accumulation: Diagnosis and Management. *J Mov Disord*. 2015;8(1):1-13. doi:10.14802/jmd.14034

35. Clement F, Devos D, Moreau C, Coubes P, Destee A, Defebvre L. Neurodegeneration with brain iron accumulation: clinical, radiographic and genetic heterogeneity and corresponding therapeutic options. *Acta Neurol Belg*. 2007;107(1):26-31.

36. Li WB, Shen NX, Zhang C, et al. Novel PANK2 Mutations in Patients With Pantothenate Kinase-Associated Neurodegeneration and the Genotype–Phenotype Correlation. *Front Aging Neurosci*. 2022;14:848919. doi:10.3389/fnagi.2022.848919

37. Chang X, Zhang J, Jiang Y, Wang J, Wu Y. Natural history and genotype‐phenotype correlation of pantothenate kinase‐associated neurodegeneration. *CNS Neurosci Ther*. 2020;26(7):754-761. doi:10.1111/cns.13294

38. Mikati MA, Yehya A, Darwish H, Karam P, Comair Y. Deep brain stimulation as a mode of treatment of early onset pantothenate kinase-associated neurodegeneration. *Eur J Paediatr Neurol*. 2009;13(1):61-64. doi:10.1016/j.ejpn.2008.01.006

39. Lim BC, Ki CS, Cho A, et al. Pantothenate kinase-associated neurodegeneration in Korea: recurrent R440P mutation in PANK2 and outcome of deep brain stimulation: Pantothenate kinase-associated neurodegeneration in Korea. *Eur J Neurol*. 2012;19(4):556-561. doi:10.1111/j.1468-1331.2011.03589.x

40. Akcakaya NH, Iseri SU, Bilir B, et al. Clinical and genetic features of PKAN patients in a tertiary centre in Turkey. *Clin Neurol Neurosurg*. 2017;154:34-42. doi:10.1016/j.clineuro.2017.01.011

41. Artusi CA, Dwivedi A, Romagnolo A, et al. Differential response to pallidal deep brain stimulation among monogenic dystonias: systematic review and meta-analysis. *J Neurol Neurosurg Psychiatry*. 2020;91(4):426-433. doi:10.1136/jnnp-2019-322169

42. Herzog R, Weissbach A, Bäumer T, Münchau A. Complex dystonias: an update on diagnosis and care. *J Neural Transm*. 2021;128(4):431-445. doi:10.1007/s00702-020-02275-y

43. Speelman JD, Contarino MF, Schuurman PR, Tijssen MAJ, De Bie RMA. Deep brain stimulation for dystonia: patient selection and outcomes: Deep brain stimulation for dystonia. *Eur J Neurol*. 2010;17:102-106. doi:10.1111/j.1468-1331.2010.03060.x

44. Schneider SA, Bhatia KP. Rare Causes of Dystonia Parkinsonism. *Curr Neurol Neurosci Rep*. 2010;10(6):431-439. doi:10.1007/s11910-010-0136-0

45. Kim J, Shin H, Youn J, Ki CS, Cho AR, Cho JW. A Novel *PANK2* Gene Mutation with Sudden-Onset Dystonia. *Can J Neurol Sci J Can Sci Neurol*. 2012;39(3):395-397. doi:10.1017/S0317167100013597

46. Guo Y pei, Tang B sha, Guo J feng. PLA2G6-Associated Neurodegeneration (PLAN): Review of Clinical Phenotypes and Genotypes. *Front Neurol*. 2018;9:1100. doi:10.3389/fneur.2018.01100

47. Magrinelli F, Mehta S, Di Lazzaro G, et al. Dissecting the Phenotype and Genotype of *PLA2G6* ‐Related Parkinsonism. *Mov Disord*. 2022;37(1):148-161. doi:10.1002/mds.28807

48. Jia F, Fellner A, Kumar KR. Monogenic Parkinson’s Disease: Genotype, Phenotype, Pathophysiology, and Genetic Testing. *Genes*. 2022;13(3):471. doi:10.3390/genes13030471

49. Sina F, Shojaee S, Elahi E, Paisán-Ruiz C. R632W mutation in *PLA2G6* segregates with dystonia-parkinsonism in a consanguineous Iranian family. *Eur J Neurol*. 2009;16(1):101-104. doi:10.1111/j.1468-1331.2008.02356.x

50. Paisan-Ruiz C, Bhatia KP, Li A, et al. Characterization of PLA2G6 as a locus for dystonia-parkinsonism. *Ann Neurol*. 2008;65(1):19-23. doi:10.1002/ana.21415

51. Cif L, Kurian MA, Gonzalez V, et al. Atypical *PLA2G6* -Associated Neurodegeneration: Social Communication Impairment, Dystonia and Response to Deep Brain Stimulation. *Mov Disord Clin Pract*. 2014;1(2):128-131. doi:10.1002/mdc3.12030

52. Illingworth MA, Meyer E, Chong WK, et al. PLA2G6-associated neurodegeneration (PLAN): Further expansion of the clinical, radiological and mutation spectrum associated with infantile and atypical childhood-onset disease. *Mol Genet Metab*. 2014;112(2):183-189. doi:10.1016/j.ymgme.2014.03.008

53. Ozes B, Karagoz N, Schüle R, et al. PLA2G6 mutations associated with a continuous clinical spectrum from neuroaxonal dystrophy to hereditary spastic paraplegia. *Clin Genet*. 2017;92(5):534-539. doi:10.1111/cge.13008

54. Chen YJ, Chen YC, Dong HL, et al. Novel PLA2G6 mutations and clinical heterogeneity in Chinese cases with phospholipase A2-associated neurodegeneration. *Parkinsonism Relat Disord*. 2018;49:88-94. doi:10.1016/j.parkreldis.2018.02.010

55. Darling A, Aguilera-Albesa S, Tello CA, et al. PLA2G6-associated neurodegeneration: New insights into brain abnormalities and disease progression. *Parkinsonism Relat Disord*. 2019;61:179-186. doi:10.1016/j.parkreldis.2018.10.013

56. Lu CS, Lai SC, Wu RM, et al. PLA2G6 mutations in PARK14-linked young-onset parkinsonism and sporadic Parkinson’s disease. *Am J Med Genet B Neuropsychiatr Genet*. 2012;159B(2):183-191. doi:10.1002/ajmg.b.32012

57. Roze E, Lang AE, Vidailhet M. Myoclonus-dystonia: classification, phenomenology, pathogenesis, and treatment. *Curr Opin Neurol*. 2018;31(4):484-490. doi:10.1097/WCO.0000000000000577

58. Valente EM, Edwards MJ, Mir P, et al. The epsilon-sarcoglycan gene in myoclonic syndromes. *Neurology*. 2005;64(4):737-739. doi:10.1212/01.WNL.0000151979.68010.9B

59. Carecchio M, Magliozzi M, Copetti M, et al. Defining the Epsilon-Sarcoglycan (SGCE) Gene Phenotypic Signature in Myoclonus-Dystonia: A Reappraisal of Genetic Testing Criteria: Reappraisal of *SGCE* Genetic Testing Criteria. *Mov Disord*. 2013;28(6):787-794. doi:10.1002/mds.25506

60. Klein C. Myoclonus and Myoclonus-Dystonias. In: *Genetics of Movement Disorders*. Elsevier; 2003:451-471. doi:10.1016/B978-012566652-7/50042-3

61. Grünewald A, Djarmati A, Lohmann-Hedrich K, et al. Myoclonus-dystonia: significance of large *SGCE* deletions. *Hum Mutat*. 2008;29(2):331-332. doi:10.1002/humu.9521

62. Asmus F, Salih F, Hjermind LE, et al. Myoclonus-dystonia due to genomic deletions in the epsilon-sarcoglycan gene. *Ann Neurol*. 2005;58(5):792-797. doi:10.1002/ana.20661

63. Hjermind LE, Werdelin LM, Eiberg H, Krag-Olsen B, Dupont E, Sorensen SA. A novel mutation in the -sarcoglycan gene causing myoclonus-dystonia syndrome. *Neurology*. 2003;60(9):1536-1539. doi:10.1212/01.WNL.0000061480.86610.BF

64. Schule B. Genetic heterogeneity in ten families with myoclonus-dystonia. *J Neurol Neurosurg Psychiatry*. 2004;75(8):1181-1185. doi:10.1136/jnnp.2003.027177

65. Ritz K, Gerrits MCF, Foncke EMJ, et al. Myoclonus-dystonia: clinical and genetic evaluation of a large cohort. *J Neurol Neurosurg Psychiatry*. 2009;80(6):653-658. doi:10.1136/jnnp.2008.162099

66. Nardocci N, Zorzi G, Barzaghi C, et al. Myoclonus-dystonia syndrome: Clinical presentation, disease course, and genetic features in 11 families: A Clinical and Genetic Study of MDS. *Mov Disord*. 2008;23(1):28-34. doi:10.1002/mds.21715

67. Hjermind LE, Vissing J, Asmus F, et al. No muscle involvement in myoclonus-dystonia caused by ɛ-sarcoglycan gene mutations. *Eur J Neurol*. 2008;15(5):525-529. doi:10.1111/j.1468-1331.2008.02116.x

68. Saunders–Pullman R, Shriberg J, Heiman G, et al. Myoclonus dystonia: Possible association with obsessive–compulsive disorder and alcohol dependence. *Neurology*. 2002;58(2):242-245. doi:10.1212/WNL.58.2.242

69. Fuchs T, Gavarini S, Saunders-Pullman R, et al. Mutations in the THAP1 gene are responsible for DYT6 primary torsion dystonia. *Nat Genet*. 2009;41(3):286-288. doi:10.1038/ng.304

70. Bressman SB, Raymond D, Fuchs T, Heiman GA, Ozelius LJ, Saunders-Pullman R. Mutations in THAP1 (DYT6) in early-onset dystonia: a genetic screening study. *Lancet Neurol*. 2009;8(5):441-446. doi:10.1016/S1474-4422(09)70081-X

71. Lange LM, Junker J, Loens S, et al. Genotype–Phenotype Relations for Isolated Dystonia Genes: MDSGene Systematic Review. *Mov Disord*. 2021;36(5):1086-1103. doi:10.1002/mds.28485

72. Blanchard A, Ea V, Roubertie A, et al. DYT6 dystonia: Review of the literature and creation of the UMD locus-specific database (LSDB) for mutations in the THAP1 gene. *Hum Mutat*. 2011;32(11):1213-1224. doi:10.1002/humu.21564

73. Xiao J, Zhao Y, Bastian RW, et al. Novel THAP1 sequence variants in primary dystonia. *Neurology*. 2010;74(3):229-238. doi:10.1212/WNL.0b013e3181ca00ca

74. Xiromerisiou G, Houlden H, Scarmeas N, et al. THAP1 mutations and dystonia phenotypes: Genotype phenotype correlations. *Mov Disord*. 2012;27(10):1290-1294. doi:10.1002/mds.25146

75. Dauer W. Inherited Isolated Dystonia: Clinical Genetics and Gene Function. *Neurotherapeutics*. 2014;11(4):807-816. doi:10.1007/s13311-014-0297-7

76. Lohmann K, Klein C. Update on the Genetics of Dystonia. *Curr Neurol Neurosci Rep*. 2017;17(3):26. doi:10.1007/s11910-017-0735-0

77. Ozelius LJ, Hewett JW, Page CE, et al. The early-onset torsion dystonia gene (DYT1) encodes an ATP-binding protein. *Nat Genet*. 1997;17(1):40-48. doi:10.1038/ng0997-40

78. Bressman SB. Dystonia genotypes, phenotypes, and classification. *Adv Neurol*. 2004;94:101-107.

79. Fasano A, Nardocci N, Elia AE, Zorzi G, Bentivoglio AR, Albanese A. Non-DYT1 early-onset primary torsion dystonia: Comparison with DYT1 phenotype and review of the literature. *Mov Disord*. 2006;21(9):1411-1418. doi:10.1002/mds.21000

80. Bressman SB, Sabatti C, Raymond D, et al. The DYT1 phenotype and guidelines for diagnostic testing. *Neurology*. 2000;54(9):1746-1753. doi:10.1212/WNL.54.9.1746

81. Steel D, Zech M, Zhao C, et al. Loss‐of‐Function Variants in HOPS Complex Genes *VPS16* and *VPS41* Cause Early Onset Dystonia Associated with Lysosomal Abnormalities. *Ann Neurol*. 2020;88(5):867-877. doi:10.1002/ana.25879

82. Cai X, Chen X, Wu S, et al. Homozygous mutation of VPS16 gene is responsible for an autosomal recessive adolescent-onset primary dystonia. *Sci Rep*. 2016;6(1):25834. doi:10.1038/srep25834

83. Ostrozovicova M, Jech R, Steel D, et al. A Recurrent *VPS16* p.Arg187* Nonsense Variant in Early‐Onset Generalized Dystonia. *Mov Disord*. 2021;36(8):1984-1985. doi:10.1002/mds.28647

84. Pott H, Brüggemann N, Reese R, et al. Truncating *VPS16* Mutations Are Rare in Early Onset Dystonia. *Ann Neurol*. 2021;89(3):625-626. doi:10.1002/ana.25990

85. Gu X, Lin J, Hou Y, Zhang L, Shang H. De Novo Missense Mutation of *VPS16* in a Chinese Patient with Generalized Dystonia with Myoclonus. *Mov Disord Clin Pract*. 2022;9(4):551-552. doi:10.1002/mdc3.13392

86. Li LX, Jiang LT, Liu Y, et al. Mutation screening of VPS16 gene in patients with isolated dystonia. *Parkinsonism Relat Disord*. 2021;83:63-65. doi:10.1016/j.parkreldis.2020.12.014

87. Li X, Wang L, Guo Y, Wan X. Mutations in the *VPS16* Gene in 56 Early‐Onset Dystonia Patients. *Mov Disord*. 2021;36(3):780-781. doi:10.1002/mds.28540

88. Park J, Reilaender A, Petry-Schmelzer JN, et al. Transcript-Specific Loss-of-Function Variants in *VPS16* Are Enriched in Patients With Dystonia. *Neurol Genet*. 2022;8(1):e644. doi:10.1212/NXG.0000000000000644

89. Petry-Schmelzer JN, Park J, Haack TB, Visser-Vandewalle V, Barbe MT, Wunderlich G. Long-term benefit of pallidal deep brain stimulation in a patient with VPS16-associated dystonia. *Neurol Res Pract*. 2022;4(1):21. doi:10.1186/s42466-022-00185-w

90. Santos M, Massano J, Lopes AM, Brandão AF, Freixo JP, Oliveira J. Aberrant Splicing Caused by a Novel VPS16 Variant Linked to Dystonia Type 30. *neurogenetics*. Published online May 24, 2023. doi:10.1007/s10048-023-00720-0

91. Monfrini E, Avanzino L, Palermo G, et al. Dominant *VPS16* Pathogenic Variants: Not Only Isolated Dystonia. *Mov Disord Clin Pract*. 2024;11(1):87-93. doi:10.1002/mdc3.13927

92. Garavaglia B, Vallian S, Romito LM, et al. AOPEP variants as a novel cause of recessive dystonia: Generalized dystonia and dystonia-parkinsonism. *Parkinsonism Relat Disord*. 2022;97:52-56. doi:10.1016/j.parkreldis.2022.03.007

93. Hess CW, Raymond D, Aguiar PDC, et al. Myoclonus-dystonia, obsessive-compulsive disorder, and alcohol dependence in *SGCE* mutation carriers. *Neurology*. 2007;68(7):522-524. doi:10.1212/01.wnl.0000253188.76092.06

94. Danielsson A, Carecchio M, Cif L, et al. Pallidal Deep Brain Stimulation in DYT6 Dystonia: Clinical Outcome and Predictive Factors for Motor Improvement. *J Clin Med*. 2019;8(12):2163. doi:10.3390/jcm8122163

95. Romito LM, Colucci F, Zorzi G, et al. Illustration of the long-term efficacy of pallidal deep brain stimulation in a patient with PKAN dystonia. *Parkinsonism Relat Disord*. 2024;123:106977. doi:10.1016/j.parkreldis.2024.106977

96. Duga V, Giossi R, Romito LM, et al. Long-term GPi DBS in pediatric non-degenerative genetic dystonia: a cohort study and a meta-analysis. *Mov Disord*. doi:10.1002/mds.29815

97. Carecchio M, Invernizzi F, Gonzàlez‐Latapi P, et al. Frequency and phenotypic spectrum of *KMT2B* dystonia in childhood: A single‐center cohort study. *Mov Disord*. 2019;34(10):1516-1527. doi:10.1002/mds.27771

98. Lunardini F, Satolli S, Levi V, Rossi Sebastiano D, Zorzi GS. The effect of GPi-DBS assessed by gait analysis in DYT11 dystonia: a case study. *Neurol Sci*. 2024;45(1):335-340. doi:10.1007/s10072-023-07063-6

99. Romito LM, Paio F, Andreasi NG, et al. A novel GNAL pathogenic variant leading to generalized dystonia: Immediate and sustained response to globus pallidus internus deep brain stimulation. *Parkinsonism Relat Disord*. 2023;115:105833. doi:10.1016/j.parkreldis.2023.105833

100. Carecchio M, Panteghini C, Reale C, et al. Novel GNAL mutation with intra-familial clinical heterogeneity: Expanding the phenotype. *Parkinsonism Relat Disord*. 2016;23:66-71. doi:10.1016/j.parkreldis.2015.12.012

101. Levi V, Franzini A, Rinaldo S, et al. Globus pallidus internus activity during simultaneous bilateral microelectrode recordings in status dystonicus. *Acta Neurochir (Wien)*. 2021;163(1):211-217. doi:10.1007/s00701-020-04618-w

102. Gold C, Henze DA, Koch C, Buzsáki G. On the Origin of the Extracellular Action Potential Waveform: A Modeling Study. *J Neurophysiol*. 2006;95(5):3113-3128. doi:10.1152/jn.00979.2005

103. Quiroga RQ, Nadasdy Z, Ben-Shaul Y. Unsupervised Spike Detection and Sorting with Wavelets and Superparamagnetic Clustering. *Neural Comput*. 2004;16(8):1661-1687. doi:10.1162/089976604774201631

104. Moran A, Bar-Gad I. Revealing neuronal functional organization through the relation between multi-scale oscillatory extracellular signals. *J Neurosci Methods*. 2010;186(1):116-129. doi:10.1016/j.jneumeth.2009.10.024

105. Abeles M. *Corticonics: Neural Circuits of the Cerebral Cortex*. 1st ed. Cambridge University Press; 1991. doi:10.1017/CBO9780511574566

106. Giorni A, Windels F, Stratton PG, et al. Single-unit activity of the anterior Globus pallidus internus in Tourette patients and posterior Globus pallidus internus in dystonic patients. *Clin Neurophysiol*. 2017;128(12):2510-2518. doi:10.1016/j.clinph.2017.10.003

107. Ahmadi N, Constandinou TG, Bouganis CS. *Estimation of Neuronal Firing Rate Using Bayesian Adaptive Kernel Smoother (BAKS)*. Neuroscience; 2017. doi:10.1101/204818

108. Vissani M, Cordella R, Micera S, Eleopra R, Romito LM, Mazzoni A. Spatio-temporal structure of single neuron subthalamic activity identifies DBS target for anesthetized Tourette syndrome patients. *J Neural Eng*. 2019;16(6):066011. doi:10.1088/1741-2552/ab37b4

109. Lengler J, Steger A. Note on the coefficient of variations of neuronal spike trains. *Biol Cybern*. 2017;111(3-4):229-235. doi:10.1007/s00422-017-0717-y

110. Shinomoto S, Shima K, Tanji J. Differences in Spiking Patterns Among Cortical Neurons. *Neural Comput*. 2003;15(12):2823-2842. doi:10.1162/089976603322518759

111. Chacron MJ, Lindner B, Longtin A. ISI CORRELATIONS AND INFORMATION TRANSFER. *Fluct Noise Lett*. 2004;04(01):L195-L205. doi:10.1142/S0219477504001793

112. Gourévitch B, Eggermont JJ. A nonparametric approach for detection of bursts in spike trains. *J Neurosci Methods*. 2007;160(2):349-358. doi:10.1016/j.jneumeth.2006.09.024

113. Herrmann CS, Murray MM, Ionta S, Hutt A, Lefebvre J. Shaping Intrinsic Neural Oscillations with Periodic Stimulation. *J Neurosci*. 2016;36(19):5328-5337. doi:10.1523/JNEUROSCI.0236-16.2016

114. Brown P, Oliviero A, Mazzone P, Insola A, Tonali P, Di Lazzaro V. Dopamine Dependency of Oscillations between Subthalamic Nucleus and Pallidum in Parkinson’s Disease. *J Neurosci*. 2001;21(3):1033-1038. doi:10.1523/JNEUROSCI.21-03-01033.2001

115. Mureşan RC, Jurjuţ OF, Moca VV, Singer W, Nikolić D. The Oscillation Score: An Efficient Method for Estimating Oscillation Strength in Neuronal Activity. *J Neurophysiol*. 2008;99(3):1333-1353. doi:10.1152/jn.00772.2007

116. Sani S, Ostrem JL, Shimamoto S, Levesque N, Starr PA. Single unit “pauser” characteristics of the globus pallidus pars externa distinguish primary dystonia from secondary dystonia and Parkinson’s disease. *Exp Neurol*. 2009;216(2):295-299. doi:10.1016/j.expneurol.2008.12.006

117. Elias S, Joshua M, Goldberg JA, et al. Statistical properties of pauses of the high-frequency discharge neurons in the external segment of the globus pallidus. *J Neurosci Off J Soc Neurosci*. 2007;27(10):2525-2538. doi:10.1523/JNEUROSCI.4156-06.2007

118. Lettieri C, Rinaldo S, Devigili G, et al. Deep brain stimulation: Subthalamic nucleus electrophysiological activity in awake and anesthetized patients. *Clin Neurophysiol*. 2012;123(12):2406-2413. doi:10.1016/j.clinph.2012.04.027

119. Fritz CO, Morris PE, Richler JJ. Effect size estimates: Current use, calculations, and interpretation. *J Exp Psychol Gen*. 2012;141(1):2-18. doi:10.1037/a0024338

120. Cohen J. *Statistical Power Analysis for the Behavioral Sciences*. 0 ed. Routledge; 2013. doi:10.4324/9780203771587
